# Identification of Single Nucleotide Polymorphism in Red Clover (*Trifolium pratense L.*) Using Targeted Genomic Amplicon Sequencing and RNA-seq

**DOI:** 10.3389/fpls.2019.01257

**Published:** 2019-10-23

**Authors:** Wenli Li, Heathcliffe Riday, Christina Riehle, Andrea Edwards, Randy Dinkins

**Affiliations:** ^1^US Dairy Forage Research Center, USDA-ARS, Madison, WI, United States; ^2^Department of Genetics, University of Wisconsin–Madison, Madison, WI, United States; ^3^Department of Biology, University of Wisconsin–Madison, Madison, WI, United States; ^4^USDA-ARS Forage-Animal Production Research Unit, N220 Ag. Science Center, N. University of Kentucky, Lexington KY, United States

**Keywords:** red clover, single-nucleotide polymorphism, targeted amplicon sequencing, RNA-seq, self-incompatibility

## Abstract

Red clover (*Trifolium pratense L.*) is a diploid, naturally cross-pollinated, cool-season species. As a perennial forage legume, red clover is mostly cultivated in temperate regions worldwide. Being a non-model crop species, genomic resources for red clover have been underdeveloped. Thus far, genomic analysis used in red clover has mainly relied on simple sequence repeat (SSR) markers. However, SSR markers are sparse in the genome and it is often difficult to unambiguously map them using short reads generated by next generation sequencing technology. Single nucleotide polymorphisms (SNPs) have been successfully applied in genomics assisted breeding in several agriculturally important species. Due to increasing importance of legumes in forage production, there is a clear need to develop SNP based markers for red clover that can be applied in breeding applications. In this study, we first developed an analytical pipeline that can confidently identify SNPs in a set of 72 different red clover genotypes using sequences generated by targeted amplicon sequencing. Then, with the same filtering stringency used in this pipeline, we used sequences from publicly available RNA-seq data to identify confident SNPs in different red clover varieties. Using this strategy, we have identified a total of 69,975 SNPs across red clover varieties. Among these, 28% (19,116) of them are missense mutations. Using *Medicago truncatula* as the reference, we annotated the regions affected by these missense mutations. We identified 2,909 protein coding regions with missense mutations. Pathway analysis of these coding regions indicated several biological processes impacted by these mutations. Specifically, three domains (homeobox domain, pentatricopeptide repeat containing plant-like, and regulator of Vps4 activity) were identified with five or more missense SNPs. These domain might also be a functional contributor in the molecular mechanisms of self-incompatibility in red clover. Future in-depth sequence diversity analysis of these three genes may yield valuable insights into the molecular mechanism involved in self-incompatibility in red clover.

## Background

Red clover (*Trifolium pratense* L.) is a diploid, naturally pollinated, cool-season species (Quesenberry and Taylor, 1996). It belongs to the tribe Trifolieae, in the legume family. As a perennial forage legume species, red clover is mostly cultivated in temperate regions worldwide. Red clover is adapted to a wide range of soils, with the ability to fix atmospheric N through symbiosis with *Rhizobium* species (Lerue and Patterson, 1981). Red clover’s shade tolerance and ability to adapt to poor soils have made red clover a great choice for intercropping with corn silage (*Zea mays* L.) to control soil erosion (Wall et al., 1991). Similar to other legume species, red clover produces isoflavones, which function as antioxidants, phytoestrogens, and antimicrobial compounds. Among them are polyphenol oxidases (PPOs), which have been shown to protect plant proteins from degradation in rumen (Jones et al., 1995). Additionally, red clover offers high-value feed to livestock, due to its high digestibility, high voluntary intake by livestock and high protein content during ensiling (Frame et al., 1998). Specifically, red clover is suitable as a conserved or gazed feed. All these unique features make red clover one of the most important forage legume species for livestock. With the burgeoning interest in sustainable agriculture and organic farming, the use of red clover has attracted renewed interest as a cover crop, forage, and/or green manure (Taylor, 2008).

Red clover is highly self-incompatible, which leads to its extremely polymorphic genome. Consequently, natural ecotypes and varieties of red clover can appear similar morphologically, but have highly heterogeneous genetic content (Istvanek et al., 2017). Thus, genomic profiling based evaluation of genetic variation is very important for reliable identification of parentage, seed purity analysis, pasture population analysis and selection of genetically divergent varieties for genetic mapping studies (Forster, 2001). Despite its highly polymorphic genome, the potential of using genomic variation for trait mapping and informed breeding is largely untapped in red clover. Thus, identification of genomic markers are needed as the first step to a wide range of genomic diversity associated applications in red clover.

Being a non-model crop species, genomic resources for red clover have been underdeveloped. Its nuclear genome is comprised of seven chromosomes, with an estimated genome size of 418 Mbp (Vizintin et al., 2006). Early studies of genome structure in red clover were carried out using fluorescence *in situ* hybridization (Sato et al., 2005; Kataoka et al., 2012). The first consensus linkage map of red clover contained 1,414 SSRs, 181 amplified length polymorphisms and 228 restriction fragment length polymorphisms (Isobe et al., 2009). DNA markers were also used to identify quantitative loci associated with persistence, disease resistance and winter hardiness (Herrmann et al., 2008; Klimenko et al., 2010). Whole-genome sequencing and assembly have provided a great platform for advances in genomics studies into traits of agronomic importance and for molecular marker assisted breeding in red clover (Istvanek et al., 2014; De Vega et al., 2015). Transcriptome sequencing has also been used to study gene expression changes in drought stress (Yates et al., 2014; Chakrabarti et al., 2016) and leaf senescence (Chao et al., 2018a). A recently study reported the sequences and complete structure of full-length transcripts in red clover using single-molecule, long-read sequencing (Chao et al., 2018b).

By reanalyzing of previously published whole genome sequencing reads of red clover, Istvanek et al. (Istvanek et al., 2017) reported genome-wide, gene-specific SSR and SNPs. In their study, an average of 5.3 SNPs were found per gene and one SSR marker was found per 12.39 kbp in the genome, indicating the rich genetic diversity of red clover that can be used for future development of genomics-based breeding applications and identification of genetic markers for key traits (Istvanek et al., 2017).

Whole genome sequencing has been widely used in SNP identification and analysis. Typically, hundreds of thousands of SNPs are identified through genome-wide comparison between a targeted genotype and the reference genome. These SNPs might be useful in developing large-scale, genotyping-based breeding selection tools. However, the majority of identified SNPs *via* genomic sequencing analysis are located in the non-coding regions of the genome, and are most likely not under selection pressure (Shastry, 2002). Without further functional annotation (e.g., targeted variant editing analysis for coding variants), the actual functional role of the large quantity of SNPs, especially those in the non-coding regions, remains unknown, for use as trait-associated selection markers.

Transcriptome studies can help interpret the functional elements of the genome. These functional elements might contribute to various phenotypes, including characteristics of cells and tissues, developmental stages, and diseases (Shah et al., 2012; Lacar et al., 2016). Specifically, SNP markers in transcribed coding regions can provide invaluable resources for marker-assisted selection, and the development of trait-associated genetic markers. With the application of high throughput RNA-seq, researchers can now assess the expression of tens of thousands of transcripts in any given organism or tissue type in an unbiased manner. Additionally, RNA-seq is capable of providing genetic information at single-nucleotide resolution (Mortazavi et al., 2008) and the opportunity to detect allele-specific expression (Gregg et al., 2010; Zhuo et al., 2017). RNA-seq based SNP biomarker identification has led to SNP identification in coding/transcribed regions of the genome (Ulloa et al., 2015; Lopez-Maestre et al., 2016). Missense SNPs in coding regions are of particular interest as they are expected to affect the function of a protein, and therefore have a more direct functional impact (Botstein and Risch, 2003; Hirschhorn and Daly, 2005; Lopez-Maestre et al., 2016). Consequently, a number of disease-associated SNPs have been reliably identified with a larger proportion of them as missense variants (Botstein and Risch, 2003; Shah et al., 2009; Kridel et al., 2012; Shah et al., 2012).

In this study, we developed an analytical pipeline to confidently identify SNPs using reads generated by targeted amplicon sequencing. Specifically, by comparing genomic sequences of one variety of red clover, Cherokee, to published reference genome (cultivar Milvus B), we identified and confirmed a set of highly confident SNPs using targeted amplicon sequencing. Then, using the same analytical strategy, RNA-seq reads were used to identify a set of highly confidently SNPs in transcribed regions in red clover. SNPs generated from this study will contribute to further development of informative genomics tools for red clover breading studies and applications.

## Materials and Methods

### SNP Confirmation Using Targeted Amplicon Sequencing

Before performing SNP identification using RNA-seq reads, we conducted a pilot study by comparing genomic sequences of Cherokee cultivar (data not shown) with that of the draft genome of red clover (De Vega et al., 2015) downloaded from NCBI Bioproject (accession number: PRJEB9186). The main purpose of this study was to develop an analytical pipeline that can confidently identify SNPs using targeted amplicon sequencing. Paired-end, genomic reads were mapped to the red clover reference genome using BWA (Li and Durbin, 2009). Mapped reads were filtered using samtools view with “-q 10” option (Li et al., 2009) to keep uniquely mapped reads. Variants were called using samtools mpileup (Li et al., 2009). Variants with less than 15 mapped reads were excluded from further analysis. An initial set of 200 SNPs were pursued for further analysis (**Supplemental File 1**). Up- and down-stream, 120 bp sequences flanking the targeted SNP were extracted for each of the SNPs to form a 240 bp fragment for each SNP. And the 240 bp sequence fragment was used to perform genome-wide mapping search to identify the fragments with unique mapping locations in the reference genome. Additionally, SNPs with indels in their proximity (up- and down-stream 100 bp region) were excluded. A final set of 36 SNPs were identified (referred as 36 loci from here on).

To confirm the final set of SNPs, primer sets were designed for each of these 36 SNPs for targeted amplicon size of 200 bp. Genomic DNAs from a total of 72 genotypes from an experimental breeding population, C328WS, were included in the confirmation study. C328WS is a red clover breeding population developed by Dr. Riday as part of his duties at USDA-ARS (Riday et al., 2015); C328WS is currently stored and maintained at the US Dairy Forage Research Center (**Supplemental Table 3**). Genomic DNAs were extracted using an adaptation of the method of Štorchová et al. (Štorchová et al., 2000; Riday et al., 2013).

For each locus, multiplex, paired-end sequencing of targeted amplicons from all 72 genotypes was performed using Illumina MiSeq (Illumina, San Diego, USA) *via* MonsterPlex, a commercial service from FloodLight Genomics LLC (http://floodlightgenomics.com/). After sequencing, paired-end reads were mapped to the red clover reference genome using BWA (Li and Durbin, 2009). Mapped reads were filtered using samtools view with “-q 10” option (Li et al., 2009) to keep uniquely mapped reads. Variants were called using samtools mpileup (Li et al., 2009). Variants with less than 15 mapped reads were excluded from further analysis.

To test the effectiveness of the identified SNPs in differentiating the 72 red clover genotypes, cluster analysis was performed using the R package pvclust (Suzuki and Shimodaira, 2006). The SNP genotyping data matrix used for the analysis was prepared as follows: homozygous reference alleles (0/0) were coded as 0, heterozygous alleles (0/1) were coded as 1 and homozygous alternative allele (1/1) were coded as 2. The bootstrap cutoff value of 85% was used to identify the significant clades.

### SNP Identification Using RNA-Seq Data

Two RNA-seq data sets were obtained from NCBI SRA database: PRJNA287846 and PRJNA219226. The data from PRJNA287846 was done by our laboratory. For this project, leaf, root and flower tissues from red clover Kenland cultivar were used for RNA-seq (Chakrabarti et al., 2016). “Kenland” is a red clover variety registered in 1951 by the Kentucky Agricultural Experiment Station (Hollowell, 1951). It is currently stored and maintained at USDA-ARS Forage-Animal Production Research Unit, in Lexington, KY. Data from PRJNA219226 (Yates et al., 2014) was downloaded from NCBI. The data was generated from young leaf tissues from F1 pseudo-testcross derived from a single genotype from each of two varieties, Milvus and Britta. Genome reference of red clover (De Vega et al., 2015) (NCBI accession PRJEB9186) was used as reference for read mapping. After quality control check, raw reads from all RNA-seq libraries were aligned using a two-step alignment approach. First, Tophat2 (Kim et al., 2013) was used with the following settings: “-r 70 –mate-std-dec 90.” Second, unmapped reads from step one were realigned with Bowtie2 using the “–very-sensitive-local” method. SNP was identified using mpileup from samtools after filtering of uniquely mapped reads. SNPs with less than 15 mapped reads were filtered out. After filtering, 600 bp sequences (300 bp up-stream and 300 bp down-stream) flaking each SNP were extracted. Using the methods we developed for genomic amplicon sequencing, flanking fragments were further checked to assess their uniqueness in the genome along with indel screening in the surrounding region. SNPs with uniquely mapped flanking fragments and without indels in their proximity were pursued with further analysis.

### Annotation of Missense SNPs and Their Impacted Genes

Potential functional effect of the SNPs identified by RNA-seq data was predicted using SNPeff (Cingolani et al., 2012). We focused on SNPs that were predicted as missense mutations. To annotate the coding regions impacted by these missense SNPs, 600 bp flanking fragment of each missense SNPs were extracted using custom Python and shell scripts (https://github.com/WLpython19/RedClover). To ensure that these SNPs were from unique regions in the genome, a genome-wide mapping search was performed using blat (Kent, 2002). Only uniquely mapped flanking fragments were pursued with gene annotation. To identify which genes these SNP flanking fragments belong to, we first extracted nucleotide sequences from *Medicago truncatula* (Young et al., 2011) from the nucleotide collection at NCBI (ftp://ftp.ncbi.nlm.nih.gov/blast/db/FASTA/nt.gz). BLASTn (Altschul et al., 1990) was used for the annotation with nucleotide sequences from *Medicago truncatula* as the reference. BLAST hits are determined as significant when E- value is less than 1e-20.

### Confirmation of SNPs Identified Using RNA-Seq Reads

Forty SNPs were selected for PCR based Sanger sequencing. Leaf tissue from red clover cultivar Kenland was used for confirmation. Both RNA and genomic DNA were extracted from leaf tissues.

Genomic DNA was extracted from 200 mg of dry leaf tissue using methods adapted from Štorchová et al. (2000) and Riday et al. (2013) with minor modifications. For cDNA synthesis, leaf tissues from Kenland was first collected and snap-frozen in liquid nitrogen before RNA extraction. Total RNA was extracted using MasterPure Complete DNA and RNA extraction kit (Illumina, San Diego, CA, US) following manufacture’s instruction. Extracted RNA samples were treated with DNAase provided by the extraction kit. Qubit RNA BR Assay kit (Thermo Fisher Scientific, USA) was used to quantify the extracted RNA samples. Two micrograms of total RNA was used using Superscript III First-Strand synthesis system (Thermo Fisher Scientific, USA) following manufacturer’s instruction.

Targeted amplicon sequencing was performed as follows. *First*, A 600 bp flanking region (300 bp up- and down-stream of the targeted SNP) for each SNP was extracted from the reference genome (PRJEB9186). These flanking regions were used for PCR primer design. Primer pairs were designed using Primer3 (Untergasser et al., 2012) (**Supplemental Table 1**). *Second*, to amplify targeted fragments, PCR reactions were performed in a reaction volume of 12 µl. PCR reaction final concentrations were 1 X JumpStart REDTaq ReadyMix (Sigma-Aldrich, St. Louis, MO), 0.20 μM of each primer, 0.50 mM additional MgCl_2_, 0.50 M betaine (Sigma-Aldrich), and approximately 20 ng template DNA. Thermal cycling was carried out on a DNA Engine Dyad (Bio-Rad) as follows: 95°C for 1 min; 45 cycles of 95°C for 20 s, 50°C for 2 min, and 72°C for 1 min; 72°C for 10 min; and a final step of 4°C for 1 min. *Third*, for sequencing of amplified targets, PCR products were submitted to the University of Wisconsin Biotechnology Center for Sanger sequencing. Sequencing traces were viewed and aligned using CodonCode Aligner 6.0.2 (Codon Code Corporation, Centerville, MA).

## Results

### Genomic SNP Confirmation Using Targeted Genomic Amplicon Sequencing

**Figure 1:**
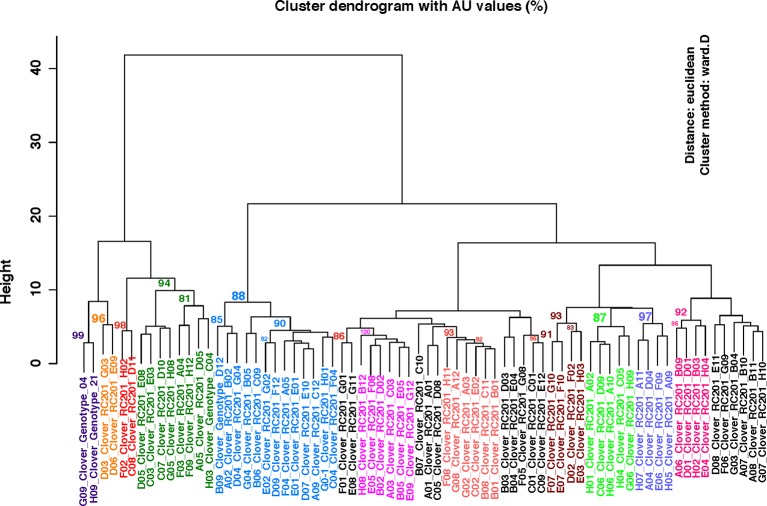
Clustering analysis using the SNP genotyping data identified using the 72 genotypes of red clover from the breeding population C328WS. Significant bootstrap values (≥85%) are indicated at each node. Randomly assigned colors are used to label the significant clades. The “Height” values on Y-axis indicate the relative distance metric between clusters.

For the pilot study, we identified a set of 36 SNPs for confirmation using targeted genomic amplicon sequencing. With the multiplex, targeted amplicon sequencing, we obtained 45,247 to 300,477 paired-end reads for each of these loci (Bioproject accession: PRJNA559390). All of the SNPs in the 36 loci were confirmed and additional nine were identified in eight of the loci (22% of the loci) (**Supplemental Table 2**). For the multiple SNPs identified from the same locus, the average spacing between them is 20 bp. In the clustering analysis, 11 subclades were identified with significant bootstrap values of 85% or more (**Figure 1**).

### SNP Confirmation and Associated Gene Annotation

**Figure 2:**
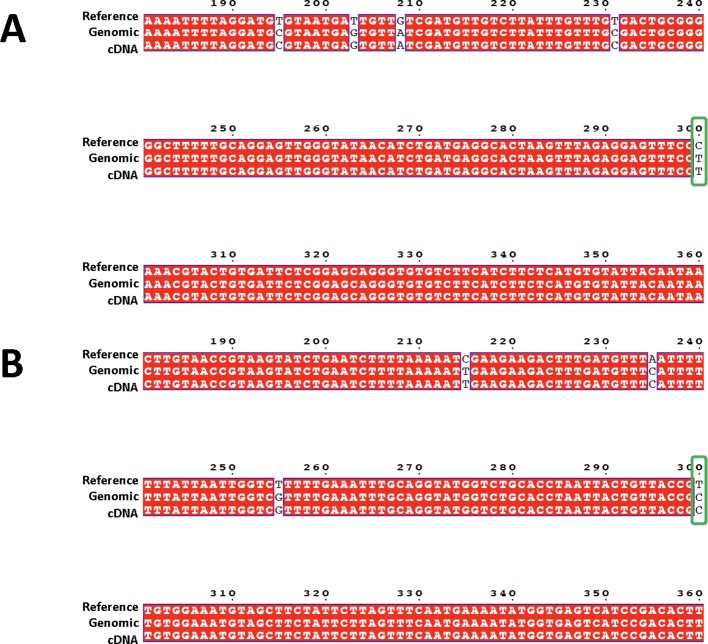
Sequence alignment showing the confirmation of target SNPs. Sequences from three sources are used for the alignment: 600-bp genome reference (top row, labeled as “Reference”, Sanger sequences generated from targeted amplicon from both genomic DNA (middle row, with “genomic” in the label”), and cDNA (bottom row, with “cDNA” in the label). Confirmed SNPs were indicated by green boxes. **(A)** is from locus A03, **(B)** is from locus A02.

A total of 69,975 confident SNPs were identified using RNA-seq data (**Supplemental File 2**). For the 40 SNPs selected for PCR based Sanger sequencing, 39 of them were confirmed by sequences obtained from both genomic DNA and cDNA (**Figure 2**, and all reads were included in **Supplemental File 3**).

Among all the identified SNPs, 28% (19,116) of them were missense mutations in protein coding regions. Annotation analysis identified 2,909 coding regions containing missense mutations. Out of these genes, 50% of them had two or more missense SNPs. Eighteen regions had the five or more missense SNPs in them (**Table 1**). A closer look at the annotated function of these genes indicated that they were associated mainly in four biological functions: 1) plan development, specifically in flower and embryo development after fertilization; 2) response to abiotic stress; 3) epigenetic control of gene expression and transcript translation; and 4) genes involved in potential mechanisms in self-incompatibility and male sterility (**Table 1**).

**Table 1:** Function annotation of top 18 protein-coding regions with missense SNPs.

Red clover missense SNP saturated genes/coding regions	Medicago Transcript	Number of missense SNPs	Annotated Function
**Serine/Threonine-kinase Nek7-like**	XM_013612047.1	5	Involved in plant development
**tRNA-specific adenosine deaminase**	XM_013599295.1	8	Key components of gene expression in every living organism
**ATP/GTP-binding family protein**	XM_003613825.2	7	Interaction with nucleotides
**BAH domain-containing protein**	XM_013595408.1	12	Plays diverse and versatile roles in chromatin biology, including protein-protein interactions, recognition of methylated histones and nucleosome binding
**RNA recognition motif (RRM) containing**	XM_013591173.1	9	Regulation of gene expression at the post-transcriptional level is mainly achieved by proteins containing well-defined sequence motifs involved in RNA binding
**calcium-dependent lipid-binding-like protein**	XM_013602890.1	9	A novel suppressor of abiotic stress response
**DCD (development and cell death) domain**	XM_013603495.1	8	Involved in development and programmed cell death; Negative regulator of cell death and defense responses. Negative regulator of several R genes, including SNC1. May have effects in promoting growth and development
**SCAR2**	XM_003623435.2	8	SCAR2 is a putative Arabidopsis WAVE complex subunit that activates the Arp2/3 complex and is required for epidermal morphogenesis
**eukaryotic aspartyl protease family**	XM_013611020.1	5	Involved in many biological processes, such as senescence, stress responses, programmed cell death, and reproduction
**ubiquitin ligase cop1, putative**	XM_013589227.1	7	RING-finger-containing protein that functions to repress plant photomorphogenesis, the light-mediated programming of plant development
**ATP synthase delta chain**	XM_003626529.2	5	
**regulator of Vps4 activity in the MVB pathway**	XM_013597625.1	8	Localized in the vacuole, presumably destined for degradation. Thus, the targeting of MVBs to the stigmatic plasma membrane or vacuole is based on whether the pollen is recognized as compatible or self-incompatible
**plant calmodulin-binding-like**	XM_003598729.2	10	These proteins are thought to be involved in various processes, such as plant defence responses and stolonisation or tuberization
**DEK carboxy-terminal domain**	XM_013604592.1	9	chromatin-associated protein
**thylakoid rhodanese-like**	XM_013589206.1	8	Associated with the inner envelope membrane, and role in photosynthetic reactions.
**E3 ubiquitin-protein ligase XBAT31, putative**	XM_013609931.1	5	E3 Ub-ligases of different families have been shown to be involved in all steps of plant immune responses.
**homeobox domain**	XM_013601504.1	5	May be required for basal embryo development after fertilization
**PPR containing plant-like**	XM_013611231.1(6)	6	Modular RNA-binding proteins which mediate several aspects of gene expression; restore male fertility, and embryogenesis

## Discussion

### Red Clover Has a Large Number of SNPs Identified in Coding/Transcribed Regions

Out of all the highly confident SNPs identified, a significant portion of them, 19,116, were missense mutations located in 2,909 annotated coding regions. Future RNA-seq analyses on additional varieties will likely identify new coding variants that could be used for functional SNP marker development. Most interestingly, 18 annotated coding regions were identified with at least five missense SNPs in them. Functional annotation of these genes revealed that they were involved in several key biological and physiological processes in plants. Of note, three annotated coding regions were identified with important roles in embryo development after fertilization (homeobox domain), potential role in cytoplasmic male sterility (PPR containing plant-like) and self-incompatibility (regulator of Vps4 activity in the MVB pathway). Homeobox domain is a DNA-binding domain of 60 amino acids, characterizing a large family of transcription factors (Mukherjee et al., 2009). This motif plays a major role in regulating gene expression of targeted genes, by recognizing and binding to specific DNA sequences. Additionally, in plants, homeobox genes have been reported as key regulators at the early stages of embryogenesis and embryonic patterning (Haecker et al., 2004; Prigge et al., 2005; Nardmann et al., 2007). PPR containing plant-like (PPR protein) is encoded by restorer (Rf) genes (Bentolila et al., 2002; Akagi et al., 2004). Rf genes are required for the development of a functional male gametophyte (Fujii et al., 2011). Studies have shown that male fertility can be restored by PPR proteins (Bentolila et al., 2002; Hanson and Bentolila, 2004). Furthermore, in *Arabidopsis*, gene knock-out studies indicated that PPR proteins were critical during early embryogenesis (Cushing et al., 2005). In *Brassicaceae*, MVBs (multi-vesicular bodies) were suggested for vesicle secretion by stigmatic papillae, which is essential for hydration and pollen tube penetration of compatible pollen grains. Thus, this protein may play a very important role in the molecular mechanisms underlying self-incompatibility (Safavian and Goring, 2013).

Red clover is known for its outcrossing nature with gametophytic self-incompatibility, which leads to high degree of genetic diversities (Riday and Krohn, 2010b;a). Such a high degree of genetic diversity imposes a substantial hurdle in genetic diversity based strategies for quantifying variation within and between populations. Identifying loci that are associated with self-incompatibility represents a vital step in tackling the highly genetically heterogeneous nature of red clover (Riday and Krohn, 2010a). For a compatible pollen to germinate and eventually produce seeds, a few steps have to happen after a pollen lands on the stigma of a flower. They include pollen germination, pollen tube growth, ovule fertilization and embryo development (Takayama et al., 2000; Charlesworth et al., 2005). A disruption at any one of these stages may contribute to self-incompatibility. Even though red clover is known for its high-degree of self-incompatibility, currently we know little about the genes instrumental for this inbreeding prevention mechanism. For the three genes/coding regions identified with high number (> = 5) missense SNPs, their annotated function indicates that they might be a functional contributor in the molecular mechanisms of self-incompatibility in red clover. Future studies might involve targeted gene capture and sequencing of these genes/domains in a larger selection of red clover varieties. Such studies will help reveal extensive degrees of genetic diversity of these genes. Additionally, they will also help provide valuable genetic markers that can be used to genetic diversity related studies and applications.

### Targeted PCR Amplification Followed by Sequencing can be a Valuable Method for Novel SNP Identification in Red Clover

As a species with high self-incompatibility, the genomic diversity of red clover deserves thorough investigation. Detailed genomic information can further facilitate breeding and molecular studies in red clover. However, genomics based diversity research is largely lacking in red clover. Our study clearly demonstrated the feasibility of targeted amplicon sequencing as an invaluable tool for genetic diversity identifications in red clover. Out of the 36 loci we pursued using targeted genomic amplicon amplification and sequencing, we discovered an additional 9 variants in 72 genotypes of red clover surveyed in the study. Taken together, 22% of the loci we investigated have additional SNPs aside from the original confirmed SNPs, with an average in-between SNPs spacing of 20 bp. Also, these 9 additional SNPs were identified based only on a 200 bp amplicon length. Our data strongly supports that red clover has a high degree of genomic variation among different cultivar varieties. Thus, it is reasonable to speculate that a large number of SNPs in red clover are still un-discovered. Currently, the most widely used genomic markers in red clover are SSR markers (Dias et al., 2008). These markers are generally scarce in the genome. Additionally, due to their repetitive nature, it is generally not easy to sequence them directly in the genome using short read technology. As evidenced by our study, targeted, amplicon-based sequencing can be used to rapidly discover novel SNPs among different varieties, without the complications of sequence assembly using a typical, whole genome sequencing approach.

Targeted amplicon sequencing based SNP identification requires the selection of a known set of loci. Once the targeted loci are selected, targeted amplicon sequencing based method has the advantage of surveying large germplasm collections from the same species for the same set of loci without the need of *de novo* primer design as shown in our study. On the other hand, two restriction enzyme digestion based methods, Multiplex restriction amplicon sequencing (MRASeq) (Bernardo et al., 2019) and genotyping by sequencing (GBS), are also used for SNP identification. GBS is a proprietary technology that requires high license and future royalty fees when used to develop commercial cultivars. Currently, the per sample GBS cost is ∼$20–$30. In comparison to GBS, MRASeq only uses two PCR steps to construct the library, omitting enzyme digestion and adapter ligation steps employed in GBS, and its estimated per sample cost is $8. However, MRASeq only amplifies a subset of restriction sites targeted by GBS. Thus, MRASeq generates fewer SNPs than GBS (Bernardo et al., 2019).

Newly identified SNPs should provide new opportunities for red clover genome research, including informed genomics assisted breeding with high density SNP markers, and even red clover seed-bank informatics development, with each germplasm’s barcoded SNP polymorphism profile recorded. Of note, using our SNP genotyping data, we were able to identify 11 sub-clades of red clover out of the initial 72 genotypes we had surveyed. This finding indicated that low-density SNP information was effective at identifying sub-population structure in red clover. Along with phenotypic data (e.g., tolerance to heat stress, high-yield, protein-content), SNP-assisted genotype clustering analysis becomes useful in the identification of cultivars with desired economic traits. Additionally, as a non-model organism, SNP surveys across populations becomes beneficial at identifying genes with high variance, like the three domains potentially linked to self-incompatibility we identified in our study. Future in-depth, population-wide deep sequencing of these domains and their predicted interacting partners will help yield insights into the dynamics of these genes in influencing self-incompatibility.

For genome-wide SNP identification using a whole genome or whole transcriptome sequencing approach, long-read based technology might be especially beneficial for red clover. Currently, the most commonly used sequencing methods are short-read based. Short-read based sequencing technologies have transformed the fields of genetics and comparative biology. However, in the case of red clover genome sequencing, accurate identification of SNPs is likely compromised by its high levels of polymorphism, especially when divergent reads from the same genomic region are recognized as different loci, subsequently are assembled inaccurately, or when the reference genome is highly divergent from the target, causing inaccurate mapping (Schneeberger et al., 2009). With the advent of single-molecule, long-read sequencing, like Pacific Biosciences (Gordon et al., 2016) and Oxford Nanopore MinION sequencing (Schneider and Dekker, 2012), it might be necessary to employ long-read sequencing for contig assembly and SNP identification in red clover. Such long-read based application will most likely improve the contig assembly of red clover. Additionally, long-reads will help more accurate phasing of SNPs that are closely linked to each other.

## Conclusions

Red clover has a large number of SNPs identified in coding regions using RNA-seq data. Specifically, three genes/domains are identified with five or more missense SNPs. Future sequence diversity analysis of these three genes may yield valuable insights into the molecular mechanism involved in self-incompatibility in red clover. Given the highly diversified genome of red clover, targeted amplicon sequencing followed by sequencing can be an effective method for novel SNP identification in red clover.

## Data Availability Statement

The data are available at NCBI under the accession number: PRJNA559390.

## Ethics Statement

The experimental research on plants carried out in this work complies with institutional, national, and international guidelines. Plant materials used in this study were from red clover C328WS and Kenland red clover. C328WS is currently stored and maintained at the US Dairy Forage Research Center. Kenland red clover is currently stored and maintained at USDA-ARS Forage-Animal Production Research Unit, in Lexington, KY. Both C328WS and Kenland are in the public domain and requires no licenses or permissions to use.

## Author Contributions

WL, HR, and RD: conceived the research project and participated in the design of the study. CR and AE: contributed to the data acquisition and analysis of targeted amplicon sequencing. WL and HR: data analysis. WL, HR, and RD: manuscript preparation and data interpretation. All authors have read and approved the final version of the manuscript.

## Funding

WL, HR, and RD were further supported by USDA Agriculture Research Service 5090-31000-024-00D, 5090-31000-026-00-D, 5090-21000-056-00D, and 5042-21000-002-00D, respectively. CR and AE are partially supported by USDA CRIS project 5090-31000-024-00D. Any mention of trade names or products is for information purposes, as the USDA does not endorse any specific products or services.

## Conflict of Interest

The authors declare that the research was conducted in the absence of any commercial or financial relationships that could be construed as a potential conflict of interest.

## Abbreviations

PPO, polyphenol oxidases; PPR, Pentatricopeptide repeat protein; Rf, restorer genes; SNP, single nucleotide polymorphism; SSR, simple sequence repeats.

## Supplementary Material

Supplemental Table 1 Sequences of primers used for validation using genomic DNA and cDNA. doi: 10.6084/m9.figshare.9971885

Supplemental Table 2 SNPs confirmed using targeted genomic amplicon sequencing. Red clover genotypes used in the study is listed in the first column. Beginning at second column, SNPs labels were listed at the top of each column. For each SNP label, the locus is indicated by the information before the last “_” (under-score sign). The numbers right after the last “_” (under-score sign) indicate the location of identified SNPs in that locus. doi: 10.6084/m9.figshare.9971882

Supplemental Table 3 Plant materials used in targeted amplicon sequencing and confirmation of SNPs identified using RNAseq data. doi: 10.6084/m9.figshare.9971888

Supplemental File 1 Genomic sequences of initial set of 200 fragments containing SNPs identified by comparing the Cherokee genomic sequences to draft genome of red clover. The genomic sequences were from the Cherokee variety of red clover. doi: 10.6084/m9.figshare.9971891

Supplemental File 2 The vcf file of 69,975 confident SNPs identified using RNAseq data. doi: 10.6084/m9.figshare.9971876

Supplemental File 3 Sequence alignment of RNA-seq identified SNPs. The alignment for each locus included sequences from the following three sources: reference sequence (with RefSeq in the label), sequence generated using genomic DNA (with gDNA in the label), sequence generated using cDNA (with cDNA in the label). doi: 10.6084/m9.figshare.9971879

## References

[B1] Akagi H. Nakamura A. Yokozeki-Misono Y. Inagaki A. Takahashi H. Mori K. (2004). Positional cloning of the rice Rf-1 gene, a restorer of BT-type cytoplasmic male sterility that encodes a mitochondria-targeting PPR protein. Theor. Appl. Genet. 108, 1449–1457. 10.1007/s00122-004-1591-2 14968308

[B2] Altschul S. F. Gish W. Miller W. Myers E. W. Lipman D. J. (1990). Basic local alignment search tool. J. Mol. Biol. 215, 403–410. 10.1016/S0022-2836(05)80360-2 2231712

[B3] Bentolila S. Alfonso A. A. Hanson M. R. (2002). A pentatricopeptide repeat-containing gene restores fertility to cytoplasmic male-sterile plants. Proc. Natl. Acad. Sci. U. S. A. 99, 10887–10892. 10.1073/pnas.102301599 12136123 PMC125068

[B4] Bernardo A. St Amand P. Le H. Q. Su Z. Bai G. (2019). Multiplex restriction amplicon sequencing: a novel next-generation sequencing-based marker platform for high-throughput genotyping. Plant Biotechnol. J. 10.1111/pbi.13192 PMC6920337 31199572

[B5] Botstein D. Risch N. (2003). Discovering genotypes underlying human phenotypes: past successes for Mendelian disease, future approaches for complex disease. Nat. Genet. 33 Suppl, 228–237. 10.1038/ng1090 12610532

[B6] Chakrabarti M. Dinkins R. D. Hunt A. G. (2016). *De novo* transcriptome assembly and dynamic spatial gene expression analysis in red clover. Plant Genome 9. 10.3835/plantgenome2015.06.0048 27898811

[B7] Chao Y. H. Xie L. J. Yuan J. B. Guo T. Li Y. R. Z. Liu F. Q. (2018a). Transcriptome analysis of leaf senescence in red clover (*Trifolium pratense* L.). Physiol. Mol. Biol. Plants 24, 753–765. 10.1007/s12298-018-0562-z 30150852 PMC6103954

[B8] Chao Y. H. Yuan J. B. Li S. F. Jia S. Q. Han L. B. Xu L. X. (2018b). Analysis of transcripts and splice isoforms in red clover (*Trifolium pratense* L.) by single-molecule long-read sequencing. Bmc Plant Biol. 18. 10.1186/s12870-018-1534-8 PMC6258457 30477428

[B9] Charlesworth D. Vekemans X. Castric V. Glemin S. (2005). Plant self-incompatibility systems: a molecular evolutionary perspective. New Phytol. 168, 61–69. 10.1111/j.1469-8137.2005.01443.x 16159321

[B10] Cingolani P. Platts A. Wang Le L. Coon M. Nguyen T. Wang L. (2012). A program for annotating and predicting the effects of single nucleotide polymorphisms, SnpEff: SNPs in the genome of Drosophila melanogaster strain w1118; iso-2; iso-3. Fly (Austin) 6, 80–92. 10.4161/fly.19695 22728672 PMC3679285

[B11] Cushing D. A. Forsthoefel N. R. Gestaut D. R. Vernon D. M. (2005). Arabidopsis emb175 and other ppr knockout mutants reveal essential roles for pentatricopeptide repeat (PPR) proteins in plant embryogenesis. Planta 221, 424–436. 10.1007/s00425-004-1452-x 15647901

[B12] De Vega J. J. Ayling S. Hegarty M. Kudrna D. Goicoechea J. L. Ergon A. (2015). Red clover (*Trifolium pratense* L.) draft genome provides a platform for trait improvement. Sci. Rep. 5, 17394. 10.1038/srep17394 26617401 PMC4663792

[B13] Dias P.M.B. Julier B. Sampoux J.P. Barre P. Dall’Agnol M. (2008). Genetic diversity in red clover (*Trifolium pratense* L.) revealed by morphological and microsatellite (SSR) markers. Euphytica 160, 189–205. 10.1007/s10681-007-9534-z

[B14] Forster J. (2001). “Application of DNA profiling to an outbreeding forage species,” in Plant genotyping: the DNA fingerprinting of plants (Wallingford: CAB International), 299–320. 10.1079/9780851995151.0299

[B15] Frame J. Charlton J. F. L. Laidlaw A. S. (1998). Temperate Forage Legumes. CAB International.

[B16] Fujii S. Bond C. S. Small I. D. (2011). Selection patterns on restorer-like genes reveal a conflict between nuclear and mitochondrial genomes throughout angiosperm evolution. Proc. Natl. Acad. Sci. U. S. A. 108, 1723–1728. 10.1073/pnas.1007667108 21220331 PMC3029733

[B17] Gordon D. Huddleston J. Chaisson M. J. Hill C. M. Kronenberg Z. N. Munson K. M. (2016). Long-read sequence assembly of the gorilla genome. Science 352, aae0344. 10.1126/science.aae0344 27034376 PMC4920363

[B18] Gregg C. Zhang J. Weissbourd B. Luo S. Schroth G. P. Haig D. (2010). High-resolution analysis of parent-of-origin allelic expression in the mouse brain. Science 329, 643–648. 10.1126/science.1190830 20616232 PMC3005244

[B19] Haecker A. Gross-Hardt R. Geiges B. Sarkar A. Breuninger H. Herrmann M. (2004). Expression dynamics of WOX genes mark cell fate decisions during early embryonic patterning in Arabidopsis thaliana. Development 131, 657–668. 10.1242/dev.00963 14711878

[B20] Hanson M. R. Bentolila S. (2004). Interactions of mitochondrial and nuclear genes that affect male gametophyte development. Plant Cell 16 Suppl, S154–S169. 10.1105/tpc.015966 15131248 PMC2643387

[B21] Herrmann D. Boller B. Studer B. Widner F Kolliker R. (2008). Improving persistence in red clover: insights from QTL analysis and comparative phenotypic evaluation. Crop Sci. 48, 269. 10.2135/cropsci2007.03.0143

[B22] Hirschhorn J. N. Daly M. J. (2005). Genome-wide association studies for common diseases and complex traits. Nat. Rev. Genet. 6, 95–108. 10.1038/nrg1521 15716906

[B23] Hollowell E. A. (1951). Registration of varieties and strains of red clover, II. Agron. J. 43, 232. 10.2134/agronj1951.00021962004300050014x

[B24] Isobe S. Kolliker R. Hisano H. Sasamoto S. Wada T. Klimenko I. (2009). Construction of a consensus linkage map for red clover (*Trifolium pratense* L.). BMC Plant Biol. 9, 57. 10.1186/1471-2229-9-57 19442273 PMC2695442

[B25] Istvanek J. Dluhosova J. Dluhos P. Patkova L. Nedelnik J. Repkova J. (2017). Gene classification and mining of molecular markers useful in red clover (*Trifolium pratense*) Breeding. Front. Plant Sci. 8, 367. 10.3389/fpls.2017.00367 28382043 PMC5360756

[B26] Istvanek J. Jaros M. Krenek A. Repkova J. (2014). Genome assembly and annotation for red clover (*Trifolium pratense*; *Fabaceae*). Am. J. Bot. 101, 327–337. 10.3732/ajb.1300340 24500806

[B27] Jones B. A. Hatfield R. Muck R. (1995). Screening legume forages for soluble phenols, polyphenol oxidase and extract browning. J. Sci. Food Agric. 67, 109–112. 10.1002/jsfa.2740670117

[B28] Kataoka R. Hara M. Kato S. Isobe S. Sato S. Tabata S. (2012). Integration of linkage and chromosome maps of red clover (*Trifolium pratense* L.). Cytogenet. Genome Res. 137, 60–69. 10.1159/000339509 22797767

[B29] Kent W. J. (2002). BLAT–the BLAST-like alignment tool. Genome Res. 12, 656–664. 10.1101/gr.229202 11932250 PMC187518

[B30] Kim D. Pertea G. Trapnell C. Pimentel H. Kelley R. Salzberg S. L. (2013). TopHat2: accurate alignment of transcriptomes in the presence of insertions, deletions and gene fusions. Genome Biol. 14, R36. 10.1186/gb-2013-14-4-r36 23618408 PMC4053844

[B31] Klimenko I. Razgulayeva N. Gau M. Okumura K. Nakaya A. Tabata S. (2010). Mapping candidate QTLs related to plant persistency in red clover. Theor. Appl. Genet. 120, 1253–1263. 10.1007/s00122-009-1253-5 20087570 PMC2839475

[B32] Kridel R. Meissner B. Rogic S. Boyle M. Telenius A. Woolcock B. (2012). Whole transcriptome sequencing reveals recurrent NOTCH1 mutations in mantle cell lymphoma. Blood 119, 1963–1971. 10.1182/blood-2011-11-391474 22210878

[B33] Lacar B. Linker S. B. Jaeger B. N. Krishnaswami S. Barron J. Kelder M. (2016). Nuclear RNA-seq of single neurons reveals molecular signatures of activation. Nat. Commun. 7, 11022. 10.1038/ncomms11022 27090946 PMC4838832

[B34] Lerue T. A. Patterson T. G. (1981). How much nitrogen do legumes fix? Adv. Agron. 34, 15–38. 10.1016/S0065-2113(08)60883-4

[B35] Li H. Durbin R. (2009). Fast and accurate short read alignment with Burrows–Wheeler transform. Bioinformatics 25, 1754–1760. 10.1093/bioinformatics/btp324 19451168 PMC2705234

[B36] Li H. Handsaker B. Wysoker A. Fennell T. Ruan J. Homer N. (2009). The sequence alignment/map format and SAMtools. Bioinformatics 25, 2078–2079. 10.1093/bioinformatics/btp352 19505943 PMC2723002

[B37] Lopez-Maestre H. Brinza L. Marchet C. Kielbassa J. Bastien S. Boutigny M. (2016). SNP calling from RNA-seq data without a reference genome: identification, quantification, differential analysis and impact on the protein sequence. Nucleic Acids Res. 44, e148. 10.1093/nar/gkw655 27458203 PMC5100560

[B38] Mortazavi A. Williams B. A. Mccue K. Schaeffer L. Wold B. (2008). Mapping and quantifying mammalian transcriptomes by RNA-Seq. Nat. Methods 5, 621–628. 10.1038/nmeth.1226 18516045 PMC13303166

[B39] Mukherjee K. Brocchieri L. Burglin T. R. (2009). A comprehensive classification and evolutionary analysis of plant homeobox genes. Mol. Biol. Evol. 26, 2775–2794. 10.1093/molbev/msp201 19734295 PMC2775110

[B40] Nardmann J. Zimmermann R. Durantini D. Kranz E. Werr W. (2007). WOX gene phylogeny in Poaceae: a comparative approach addressing leaf and embryo development. Mol. Biol. Evol. 24, 2474–2484. 10.1093/molbev/msm182 17768306

[B41] Prigge M. J. Otsuga D. Alonso J. M. Ecker J. R. Drews G. N. Clark S. E. (2005). Class III homeodomain-leucine zipper gene family members have overlapping, antagonistic, and distinct roles in Arabidopsis development. Plant Cell 17, 61–76. 10.1105/tpc.104.026161 15598805 PMC544490

[B42] Quesenberry K. Taylor N. L. (1996). Red Clover Science. Curr. Plant Sci. Biotechnol. Agric. 28.

[B43] Riday H. Johnson D. W. Heyduk K. Raasch J. A. Darling M. E. Sandman J. M. (2013). Paternity testing in an autotetraploid alfalfa breeding polycross. Euphytica, 194, 335–349. 10.1007/s10681-013-0938-7

[B44] Riday H. Krohn A. L. (2010a). Genetic map-based location of the red clover (*Trifolium pratense* L.) gametophytic self-incompatibility locus. Theor. Appl. Genet. 121, 761–767. 10.1007/s00122-010-1347-0 20461353

[B45] Riday H. Krohn A. L. (2010b). Increasing population hybridity by restricting self-incompatibility alleles in red clover populations. Crop Sci. 50, 853–860. 10.2135/cropsci2009.05.0282

[B46] Riday H. Smith M. A. Peel M. D. (2015). A simple model for pollen-parent fecundity distributions in bee-pollinated forage legume polycrosses. Theor. Appl. Genet. 128, 1865–1879. 10.1007/s00122-015-2553-6 26105686

[B47] Safavian D. Goring D. R. (2013). Secretory activity is rapidly induced in stigmatic papillae by compatible pollen, but inhibited for self-incompatible pollen in the Brassicaceae. PLoS One 8, e84286. 10.1371/journal.pone.0084286 24386363 PMC3873414

[B48] Sato S. Isobe S. Asamizu E. Ohmido N. Kataoka R. Nakamura Y. (2005). Comprehensive structural analysis of the genome of red clover (*Trifolium pratense* L.). DNA Res. 12, 301–364. 10.1093/dnares/dsi018 16769692

[B49] Schneeberger K. Hagmann J. Ossowski S. Warthmann N. Gesing S. Kohlbacher O. (2009). Simultaneous alignment of short reads against multiple genomes. Genome Biol. 10, R98. 10.1186/gb-2009-10-9-r98 19761611 PMC2768987

[B50] Schneider G. F. Dekker C. (2012). DNA sequencing with nanopores. Nat. Biotechnol. 30, 326–328. 10.1038/nbt.2181 22491281

[B51] Shah S. P. Morin R. D. Khattra J. Prentice L. Pugh T. Burleigh A. (2009). Mutational evolution in a lobular breast tumour profiled at single nucleotide resolution. Nature 461, 809–813. 10.1038/nature08489 19812674

[B52] Shah S. P. Roth A. Goya R. Oloumi A. Ha G. Zhao Y. (2012). The clonal and mutational evolution spectrum of primary triple-negative breast cancers. Nature 486, 395–399. 10.1038/nature10933 22495314 PMC3863681

[B53] Shastry B. S. (2002). SNP alleles in human disease and evolution. J. Hum. Genet. 47, 561–566. 10.1007/s100380200086 12436191

[B54] Štorchová H. Chrtek J. Jr. Tetera M. Fitze D. Fehrer J. (2000). An improved method of DNA isolation from plants collected in the field and conserved in saturated NaCl/CTAB solution. Taxon 49, 79–84. 10.2307/1223934

[B55] Suzuki R. Shimodaira H. (2006). Pvclust: an R package for assessing the uncertainty in hierarchical clustering. Bioinformatics 22, 1540–1542. 10.1093/bioinformatics/btl117 16595560

[B56] Takayama S. Shiba H. Iwano M. Shimosato H. Che F. S. Kai N. (2000). The pollen determinant of self-incompatibility in Brassica campestris. Proc. Natl. Acad. Sci. U. S. A. 97, 1920–1925. 10.1073/pnas.040556397 10677556 PMC26537

[B57] Taylor N. (2008). A century of clover breeding developments in the United States. Crop Sci. 48, 1–13. 10.2135/cropsci2007.08.0446

[B58] Ulloa P. E. Rincon G. Islas-Trejo A. Araneda C. Iturra P. Neira R. (2015). RNA sequencing to study gene expression and SNP variations associated with growth in zebrafish fed a plant protein-based diet. Mar. Biotechnol. (NY) 17, 353–363. 10.1007/s10126-015-9624-1 25702041

[B59] Untergasser A. Cutcutache I. Koressaar T. Ye J. Faircloth B. C. Remm M. (2012). Primer3—new capabilities and interfaces. Nucleic Acids Res. 40, e115. 10.1093/nar/gks596 22730293 PMC3424584

[B60] Vizintin L. Branka J. Bohanec B. (2006). Genetic characterization of selected Trifolium species as revealed by nuclear DNA content and ITS rDNA region analysis. Plant Science 170, 859–866. 10.1016/j.plantsci.2005.12.007

[B61] Wall G. Pringle E. Sheard. R. (1991). Intercropping red clover with silage corn for soil erosion control. Can. J. Soil Sci. 71, 137–145. 10.4141/cjss91-013

[B62] Yates S. A. Swain M. T. Hegarty M. J. Chernukin I. Lowe M. Allison G. G. (2014). De novo assembly of red clover transcriptome based on RNA-Seq data provides insight into drought response, gene discovery and marker identification. BMC Genomics 15, 453. 10.1186/1471-2164-15-453 24912738 PMC4144119

[B63] Young N. D. Debelle F. Oldroyd G. E. Geurts R. Cannon S. B. Udvardi M. K. (2011). The Medicago genome provides insight into the evolution of rhizobial symbioses. Nature 480, 520–524. 10.1038/nature10625 22089132 PMC3272368

[B64] Zhuo Z. Lamont S. J. Abasht B. (2017). RNA-Seq analyses identify frequent allele specific expression and no evidence of genomic imprinting in specific embryonic tissues of chicken. Sci. Rep. 7, 11944. 10.1038/s41598-017-12179-9 28931927 PMC5607270

