# Supplementary material for: Identification of Single Nucleotide Polymorphism in Red Clover (Trifolium pratense L.) Using Targeted Genomic Amplicon Sequencing and RNA-seq
Source: Front Plant Sci. 2019 Oct 23;10:1257. doi: 10.3389/fpls.2019.01257 (PMC6820467; doi:10.3389/fpls.2019.01257)

## Locus F01

Targeted SNP is indicated by black box. Other SNPs are indicated by blue boxes.

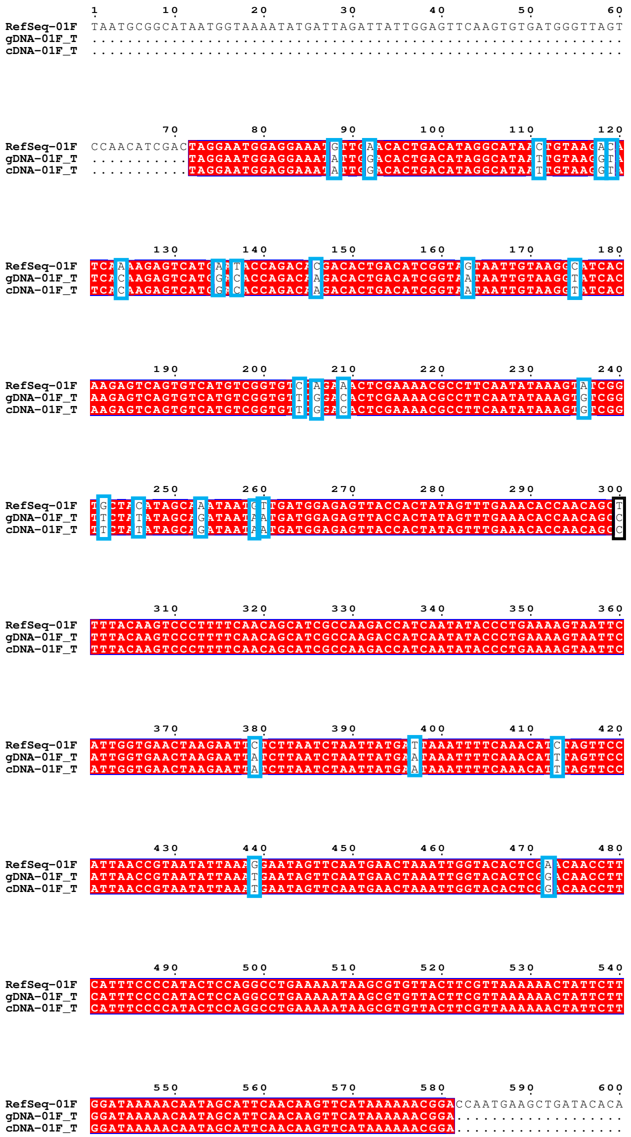

## Locus A02

Targeted SNP is indicated by black box. Other SNPs are indicated by blue boxes.

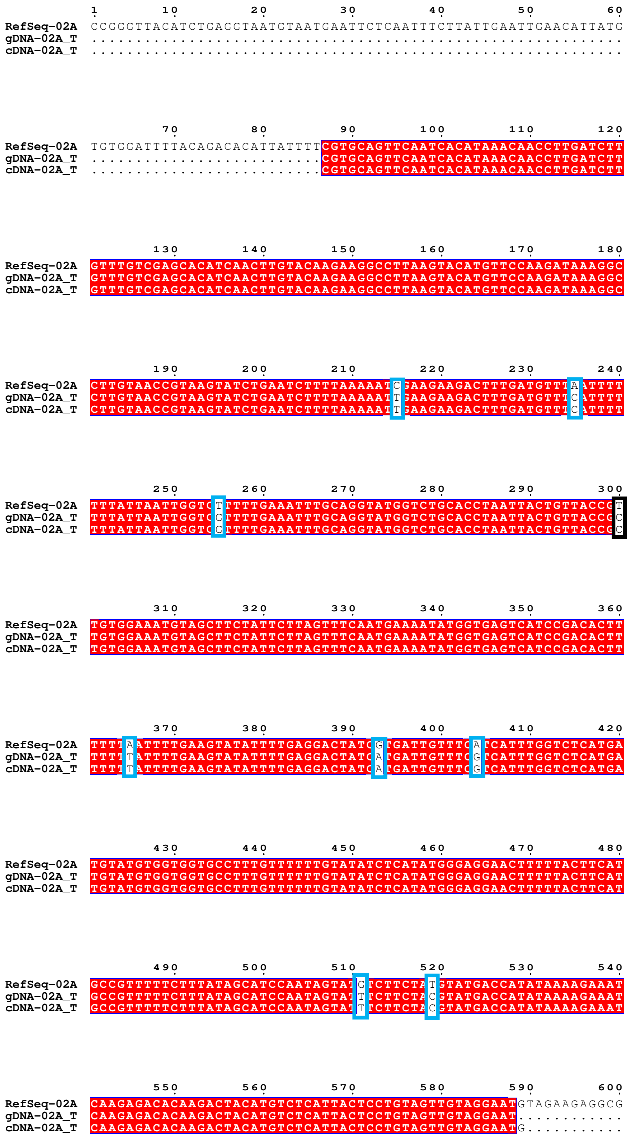

## Locus E02

Targeted SNP is indicated by black box. Other SNPs are indicated by blue boxes.

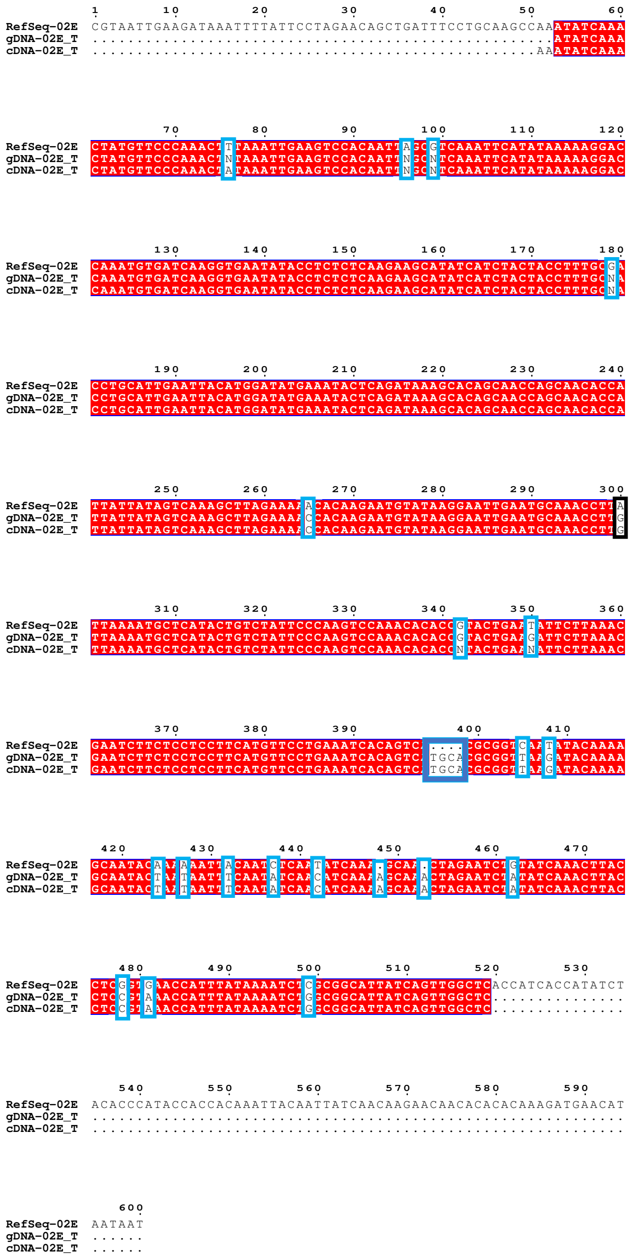

**Locus H02**

Targeted SNP is indicated by black box. Other SNPs are indicated by blue boxes.

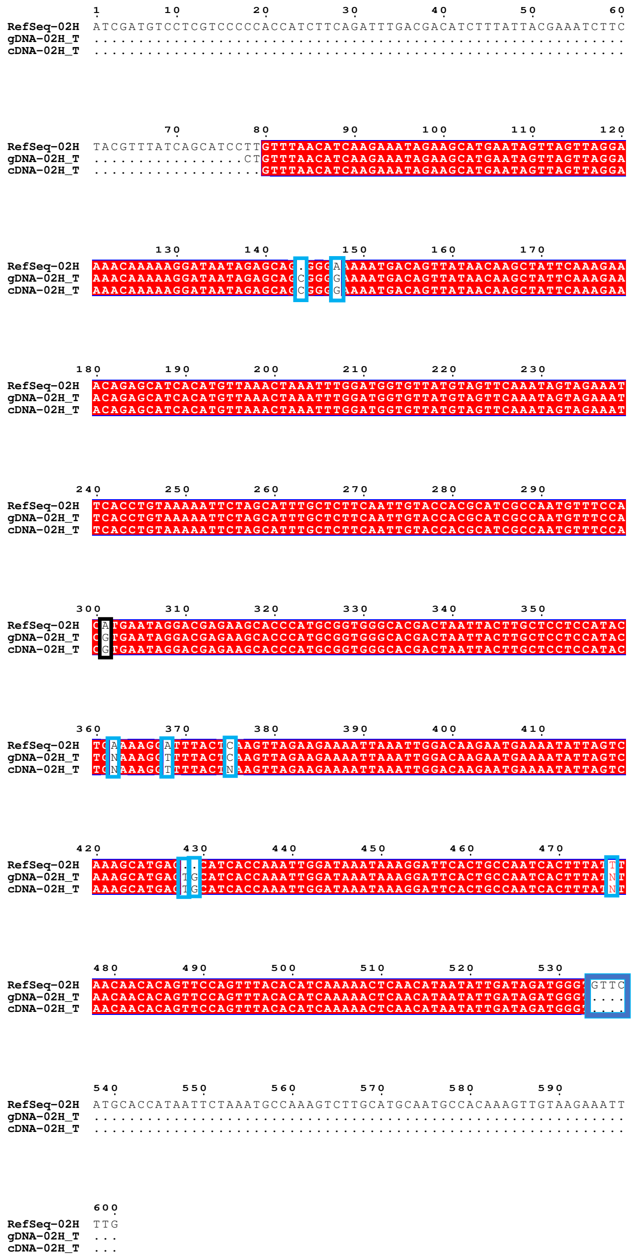

## Locus A03

Targeted SNP is indicated by black box. Other SNPs are indicated by blue boxes.

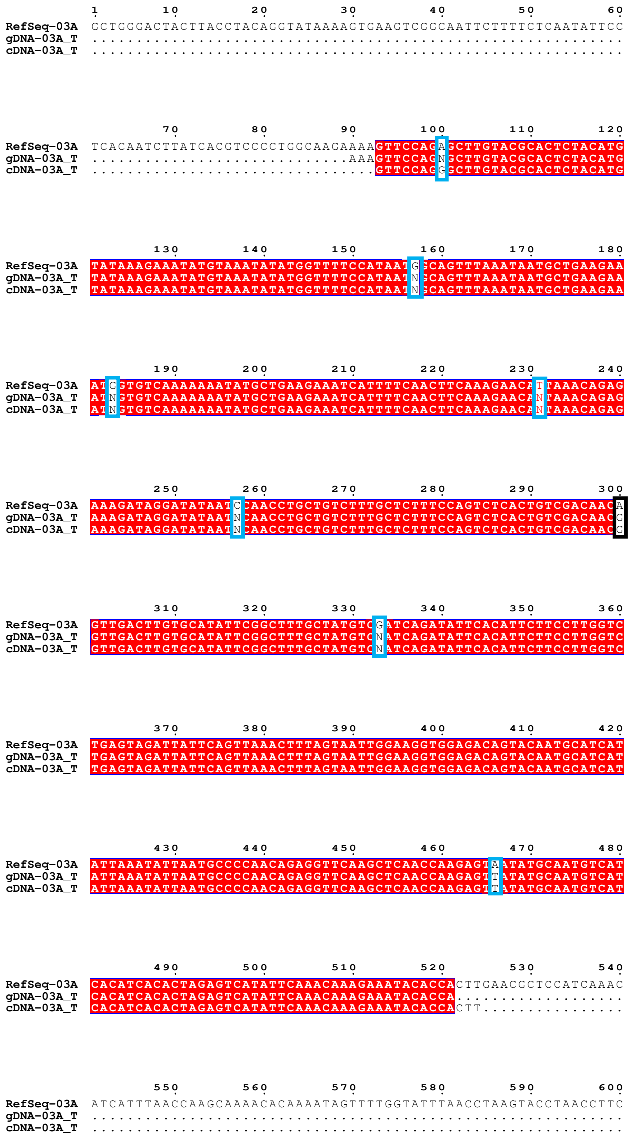

**Locus B03**

Targeted SNP is indicated by black box. Other SNPs are indicated by blue boxes.

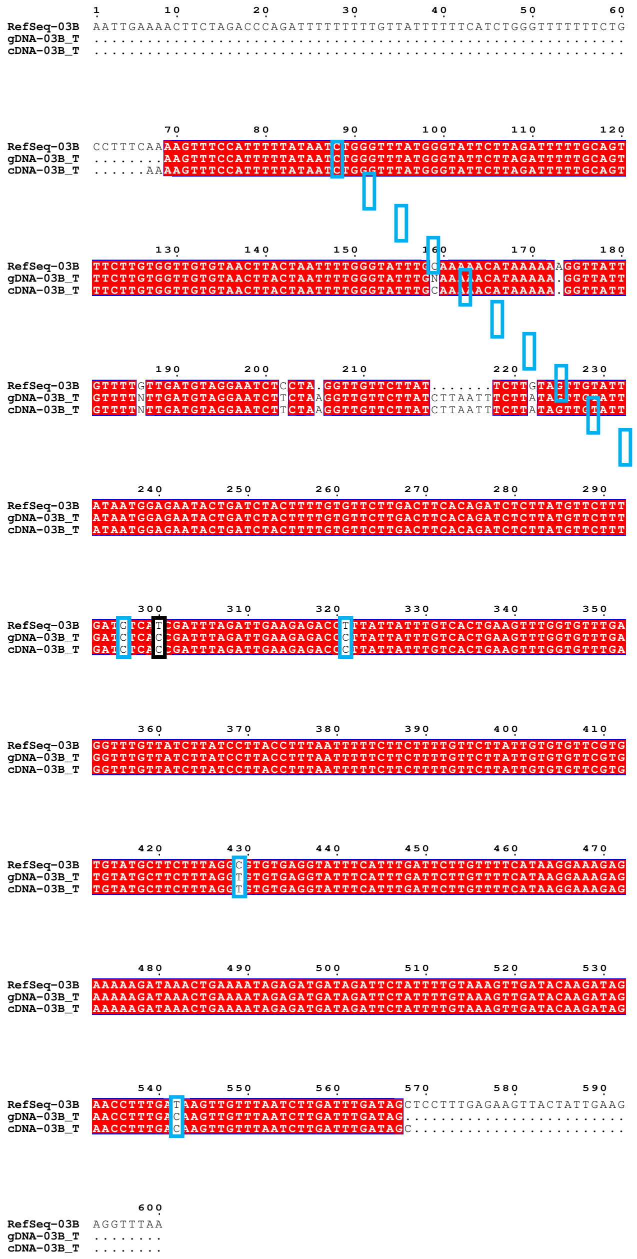

**Locus D03**

Targeted SNP is indicated by black box. Other SNPs are indicated by blue boxes.

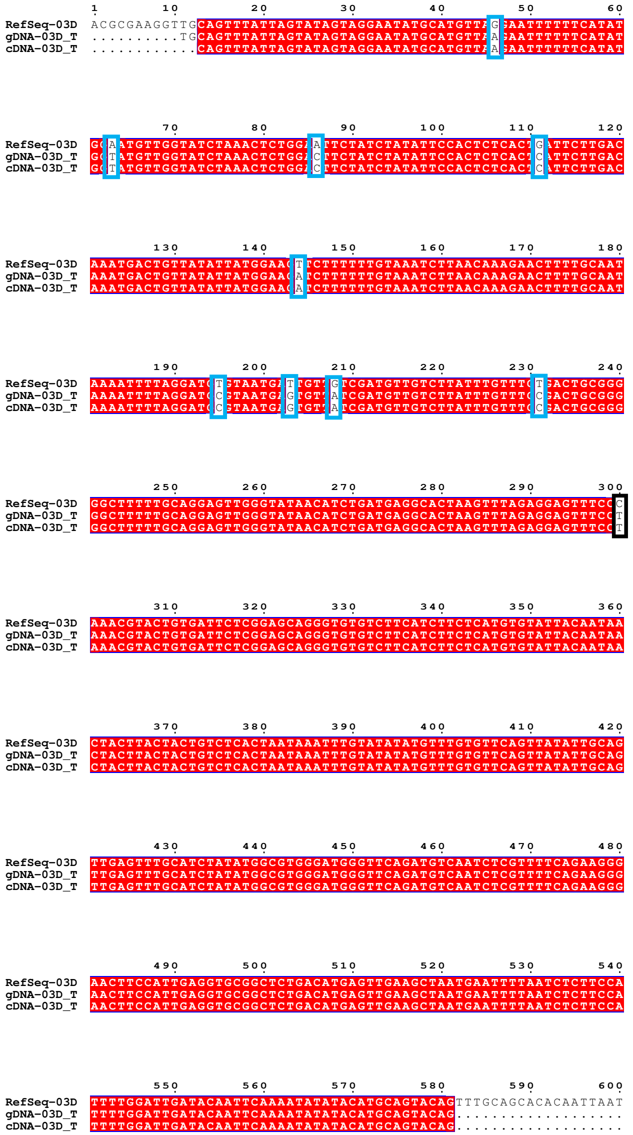

## Locus E03

Targeted SNP is indicated by black box. Other SNPs are indicated by blue boxes.

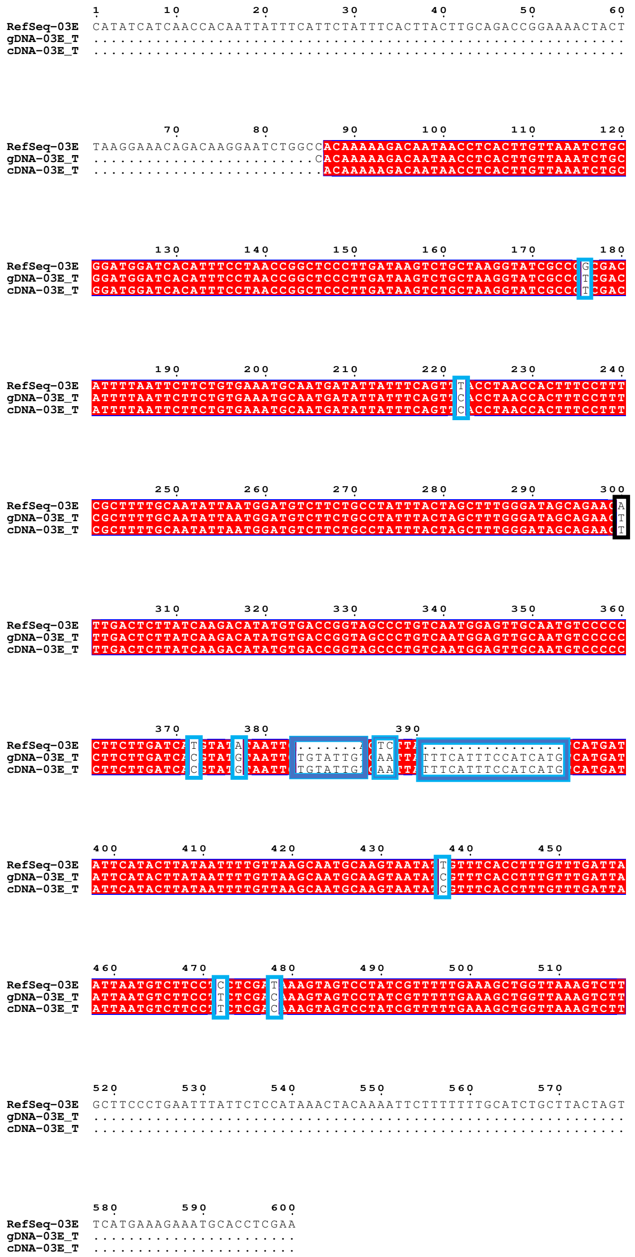

**Locus F03**

Targeted SNP is indicated by black box. Other SNPs are indicated by blue boxes.

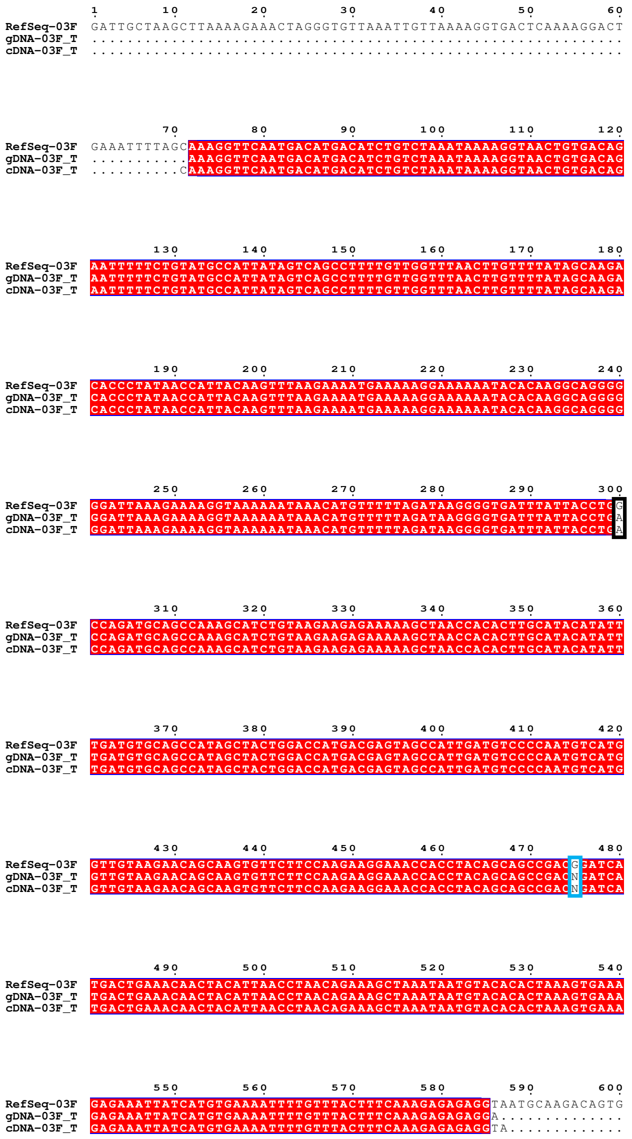

**Locus H03**

Targeted SNP is indicated by black box. Other SNPs are indicated by blue boxes.

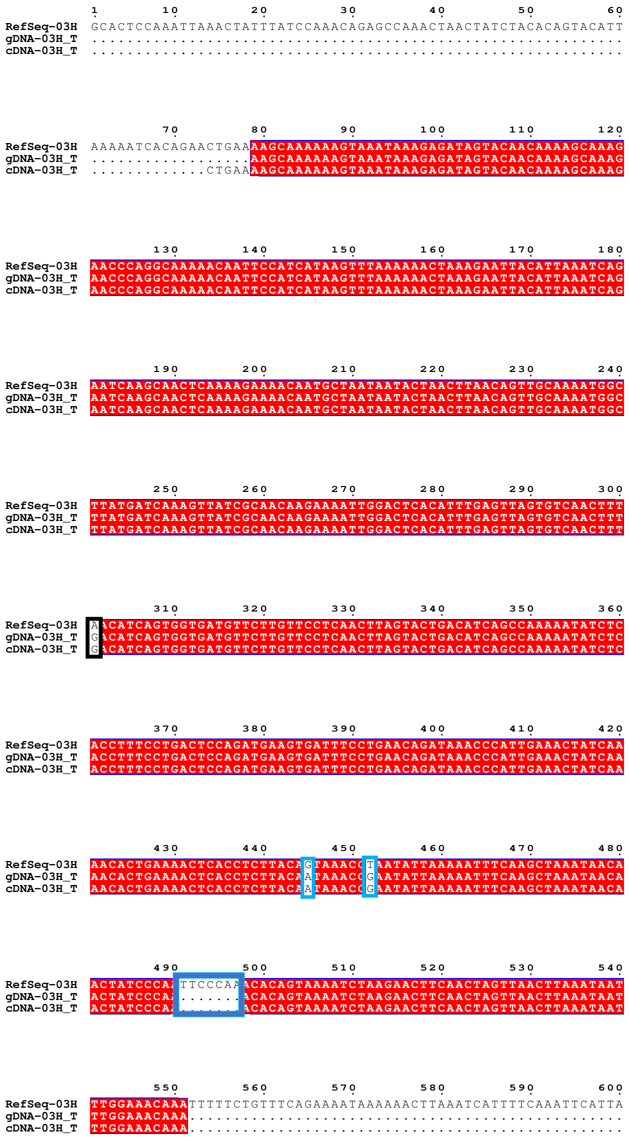

**Locus B04**

Targeted SNP is indicated by black box. Other SNPs are indicated by blue boxes.

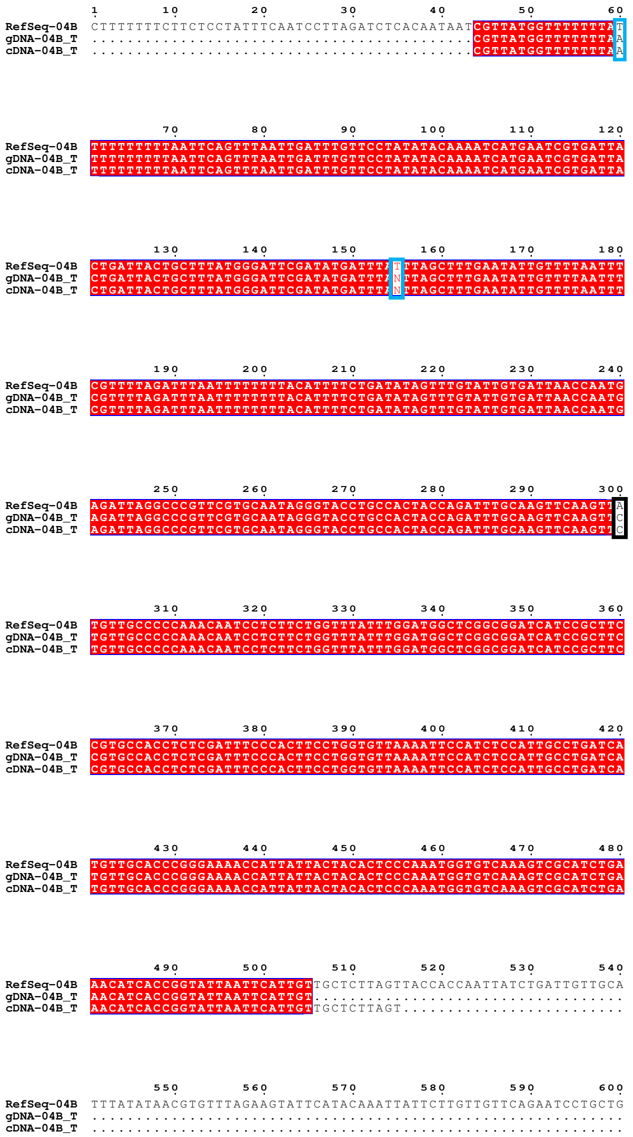

## Locus E04

Targeted SNP is indicated by black box. Other SNPs are indicated by blue boxes.

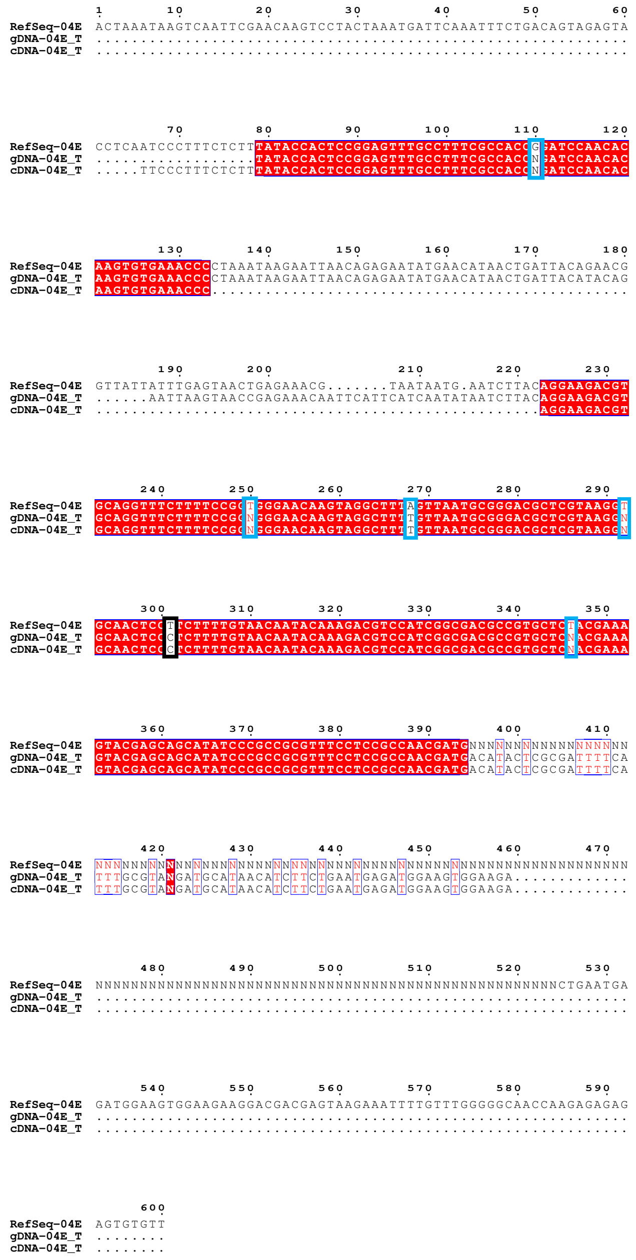

**Locus F04**

Targeted SNP is indicated by black box. Other SNPs are indicated by blue boxes.

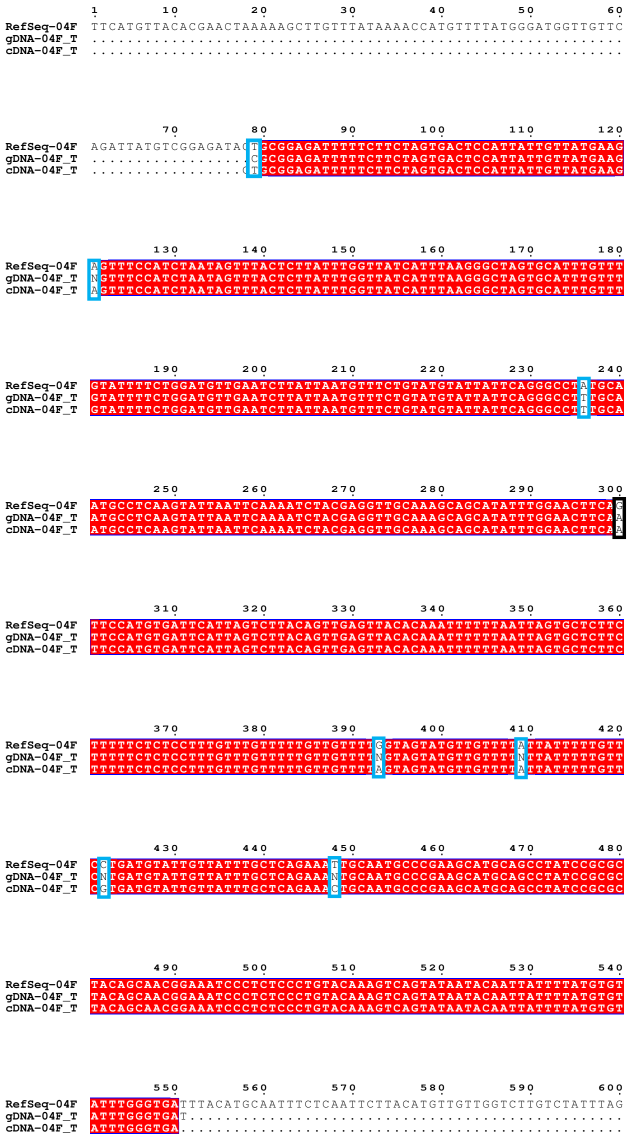

**Locus G04**

Targeted SNP is indicated by black box. Other SNPs are indicated by blue boxes.

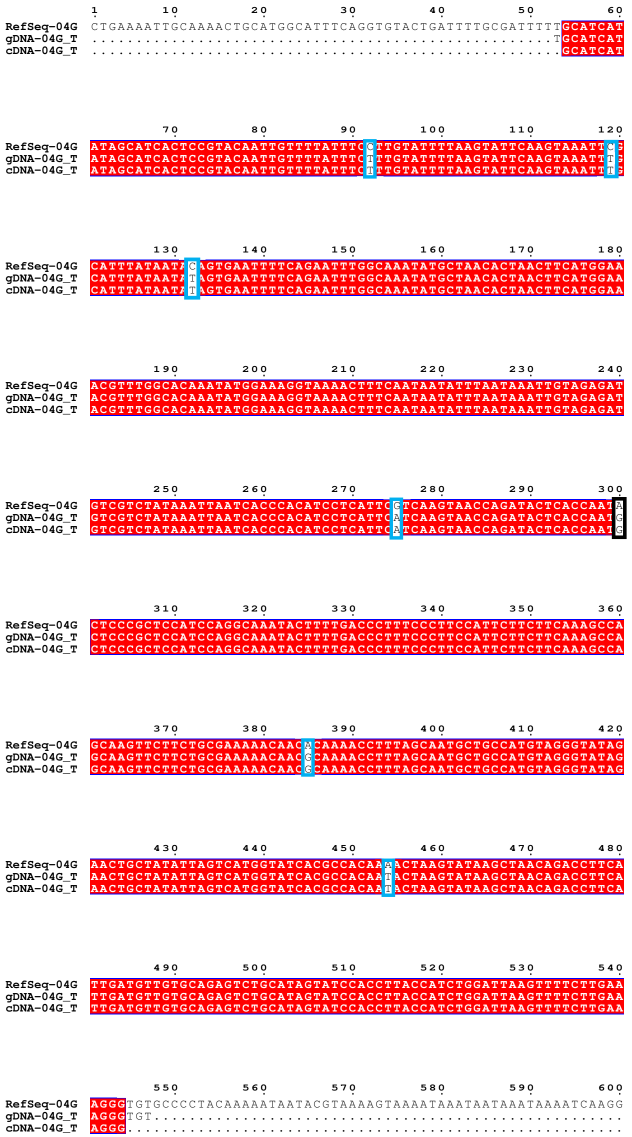

# Locus H04

Targeted SNP is indicated by black box. Other SNPs are indicated by blue boxes.

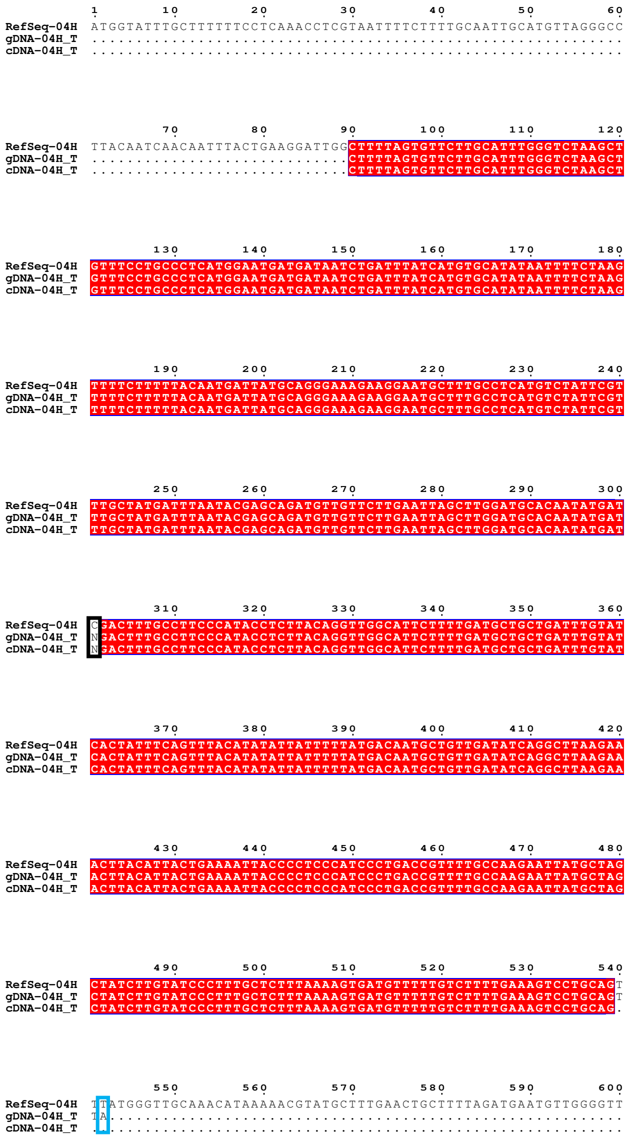

## Locus D05

Targeted SNP is indicated by black box. Other SNPs are indicated by blue boxes.

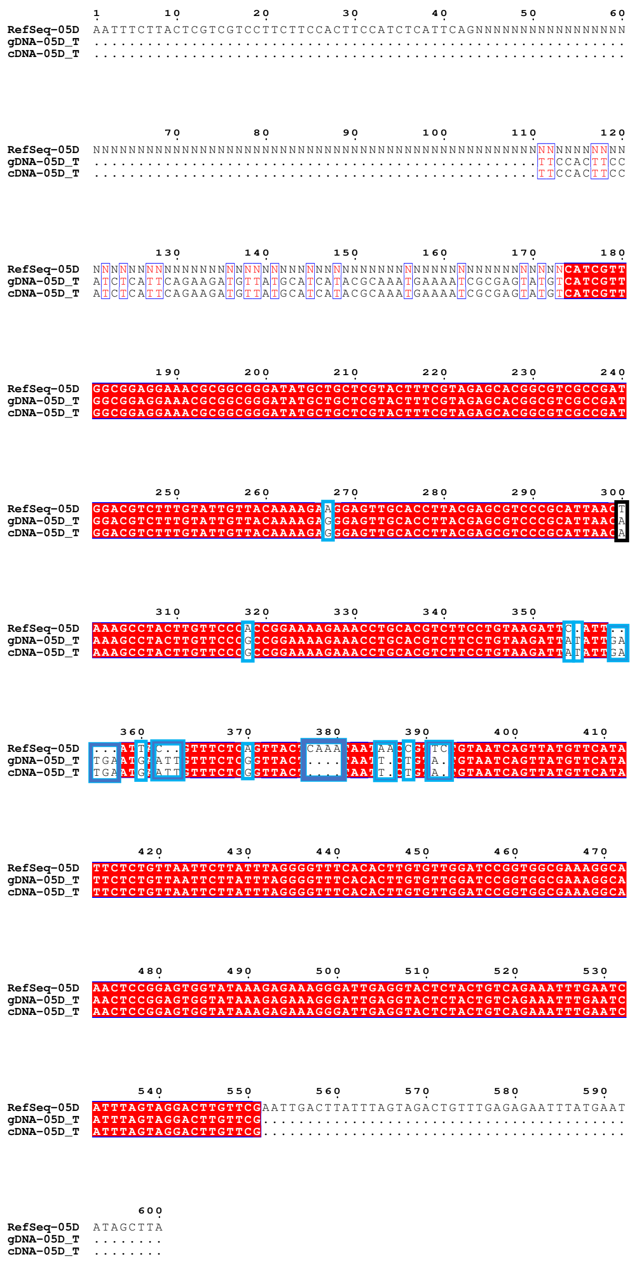

## Locus E05

Targeted SNP is indicated by black box. Other SNPs are indicated by blue boxes.

```
             1        10        20        30        40        50        60
RefSeq-05E   CACTAAACGATAGAAGAGATCTCCCAAACGATGTTTGATGACGGTGTAAGTTATCTTCTG
gDNA-05E_T   ............................................................
cDNA-05E_T   ............................................................

                      70        80        90       100       110       120
RefSeq-05E   ACCATCAGTACCAGCTCCCCTCTCTACTGCCTGCATGTAACCAGTCTTACGTTAAAGATC
gDNA-05E_T   .............GCTCCCCTCTCTACTGCCTGCATGTAACCAGTCTTACGTTAAAGATC
cDNA-05E_T   ..............CTCCCCTCTCTACTGCCTG...........................

                     130       140       150       160       170       180
RefSeq-05E   TACAATTTGCGTGCAATTTAGCCATCTTACTGAAATCTTTTTAGGCTGAAGTAATCATAC
gDNA-05E_T   TACAATTTGTGTGCAATTTAGCCATCTTACTGAAATCTTTTTAGGCTGAAGTAATCATAC
cDNA-05E_T   ............................................................

                     190       200       210       220       230       240
RefSeq-05E   CCTTTGACCGAGTTATGACAAAGAATGCAAAACATAAAGAAGAAAACAAAACATACCTGA
gDNA-05E_T   CCTTTGACCGAGTTATGACAAAGAATGCAAAACATAAAGAAGAAAACAAAACATACCTGA
cDNA-05E_T   ...........................................................A

                     250       260       270       280       290       300
RefSeq-05E   TTTGCCAAATTGTAGAAATGAATAATGTTGCGCATCATCCAGACAGACTTGTAAAAGGGA
gDNA-05E_T   TTTGCCAAATTGTAGAAATGAATAATGTTGCGCATCATCCAGACAGACTTGTAAAAGGGG
cDNA-05E_T   TTTGCCAAATTGTAGAAATGAATAATGTTGCGCATCATCCAGACAGACTTGTAAAAGGGG

                     310       320       330       340       350       360
RefSeq-05E   CAGAACTTGTCATATCTATTACACATAGAAAAGGTAACAATATAAGAGAGACAACAAGTA
gDNA-05E_T   CAGAACTTGTCATATCTATTACACATACAAAAGGTAACAATATAAGAGAGACAACAAGTA
cDNA-05E_T   CAGAACTTGTCATAT.............................................

                     370       380       390       400       410
RefSeq-05E   AAAACACTAACATGAGTCATGATGTATCAAAGAAGTTAACAATTTACAAGGGCACTTA.C
gDNA-05E_T   AAAACACTAACATGAGTCATGATGTATCAAAGAAGTTAACAATTTACAAGGGCACTTAGC
cDNA-05E_T   ............................................................

           420       430       440       450       460       470
RefSeq-05E   GGTGTAAAAGCATTTTGAGCAAGGTAATCCTCTCTCAAAAGTTTTGCAGTTTCTAGGGTT
gDNA-05E_T   GGTGTAAAAGCATTTTGAGCAAGGTAATCCTCTCTCAAAAGTTTTGCAGTTTCTAGGGTT
cDNA-05E_T   GGTGTAAAAGCATTTTGAGCAAGGTAATCCTCTCTCAAAAGTTTTGCAGTTTCTAGGGTT

           480       490       500       510       520       530
RefSeq-05E   ATCTTATCTCCTTCAGCTAAAGCATCCTTTCCTACAAGCTGGAATGAACAAGGTAGGTGA
gDNA-05E_T   ATCTTATCTCCTTCAGCTAAAGCATCCTTTC.............................
cDNA-05E_T   ATCTTATCTCCTTCAGCTAAAGCATCCTTTCCTACAAGCTGGAA................

           540       550       560       570       580       590
RefSeq-05E   ATGACATATATAGATTACTACTTTTCTACTTAGAGATTAACATTCCTGACAAATTTTGTC
gDNA-05E_T   ............................................................
cDNA-05E_T   ............................................................

           600
RefSeq-05E   C
gDNA-05E_T   .
cDNA-05E_T   .
```

**Locus A06**

Targeted SNP is indicated by black box. Other SNPs are indicated by blue boxes.

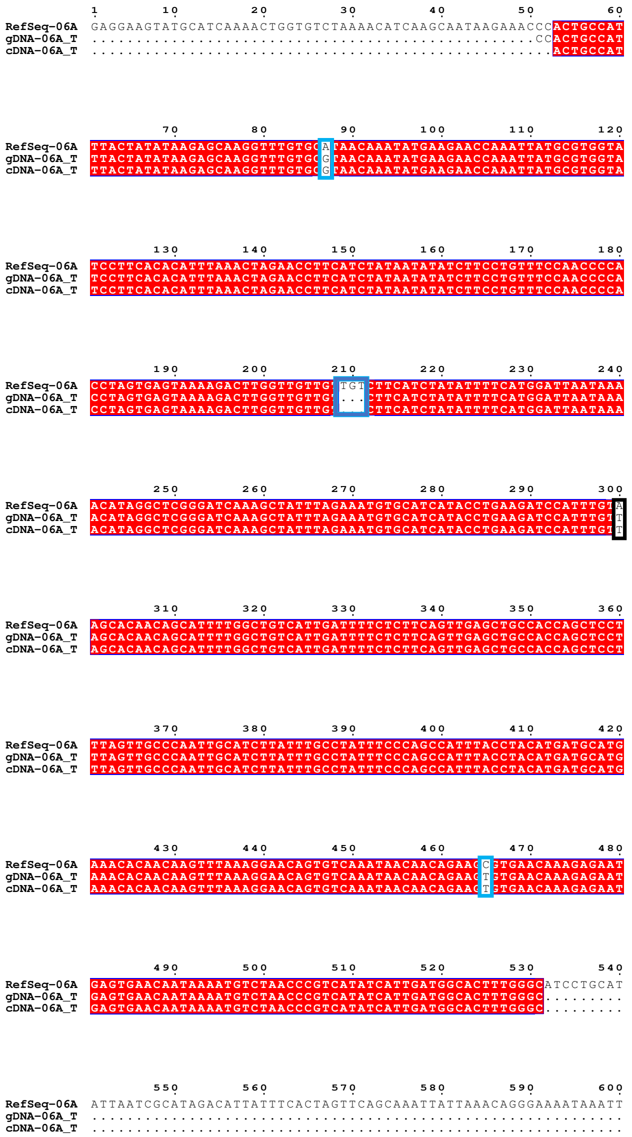

## Locus E06

Targeted SNP is indicated by black box. Other SNPs are indicated by blue boxes.

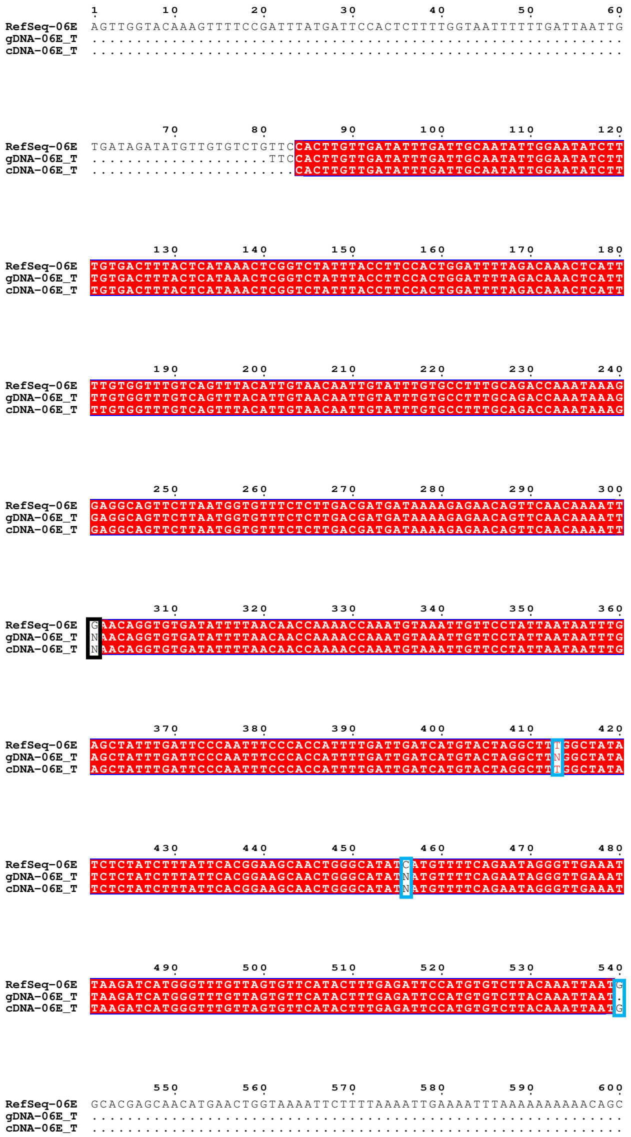

**Locus A07**

Targeted SNP is indicated by black box. Other SNPs are indicated by blue boxes.

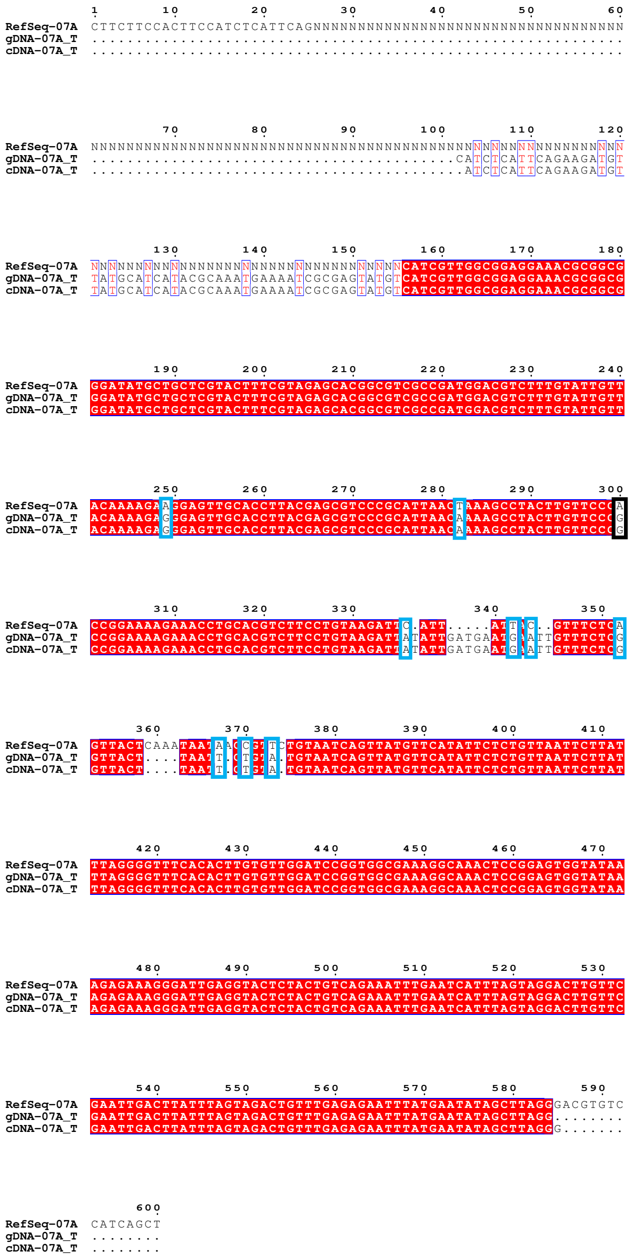

## Locus E07

Targeted SNP is indicated by black box. Other SNPs are indicated by blue boxes.

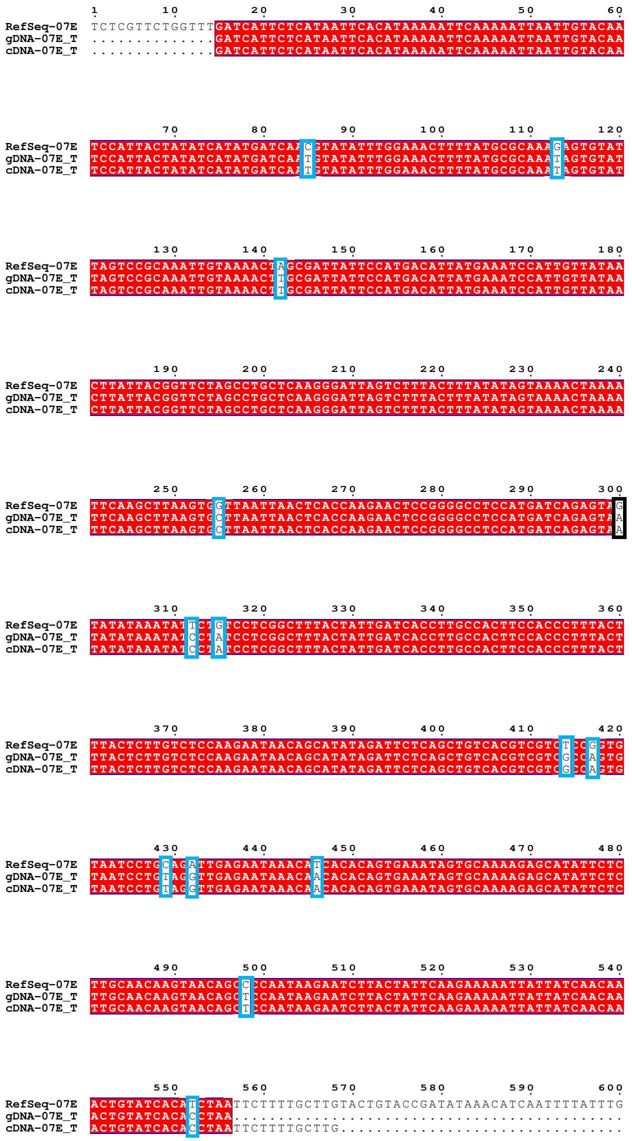

**Locus F07**

Targeted SNP is indicated by black box. Other SNPs are indicated by blue boxes.

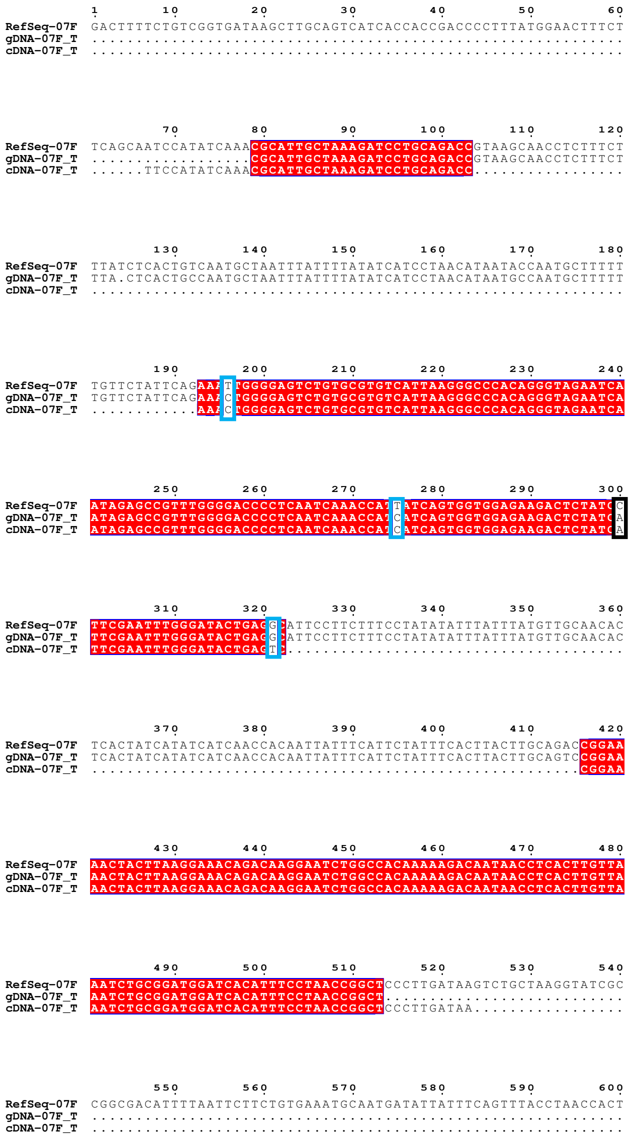

**Locus B08**

Targeted SNP is indicated by black box. Other SNPs are indicated by blue boxes.

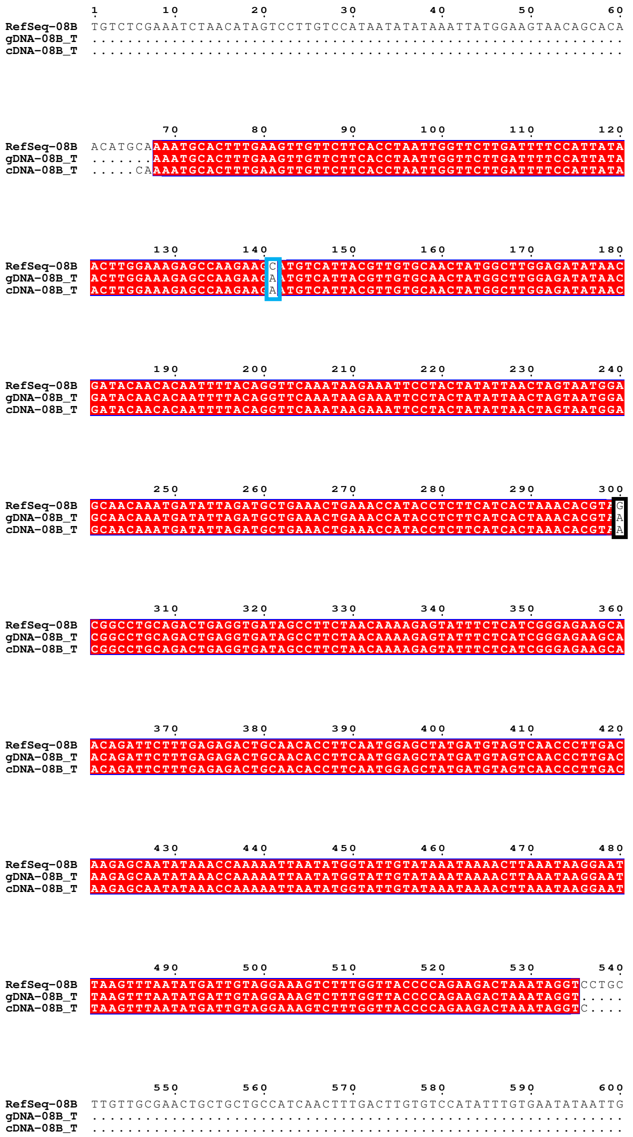

**Locus E08**

Targeted SNP is indicated by black box. Other SNPs are indicated by blue boxes.

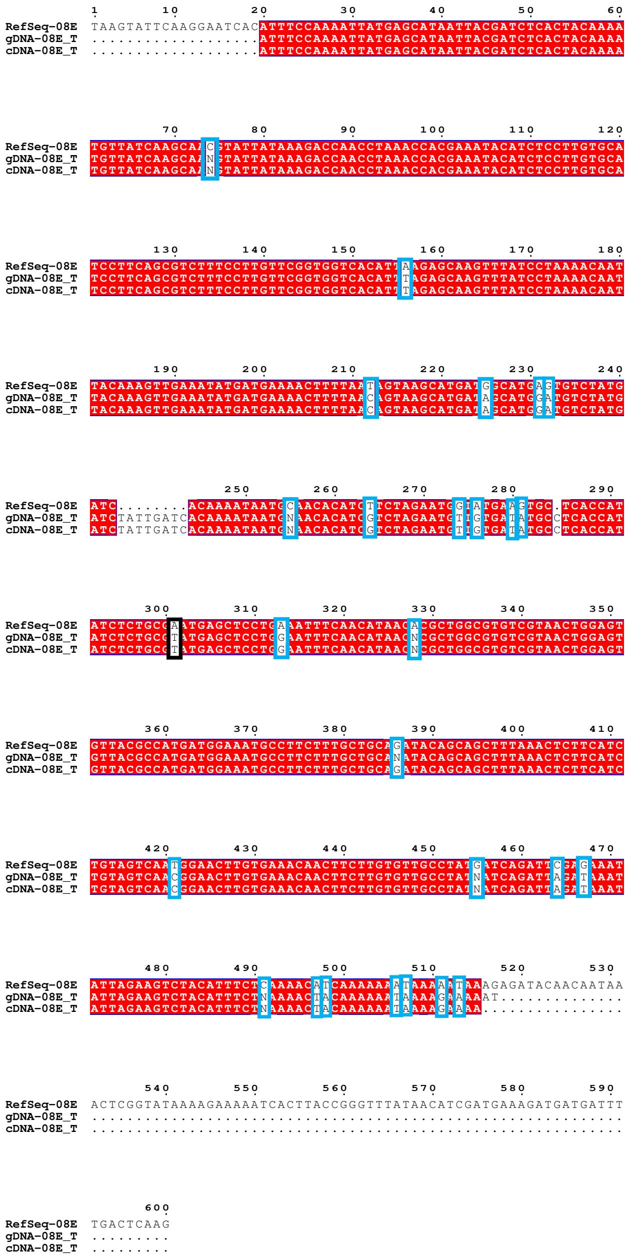

**Locus G08**

Targeted SNP is indicated by black box. Other SNPs are indicated by blue boxes.

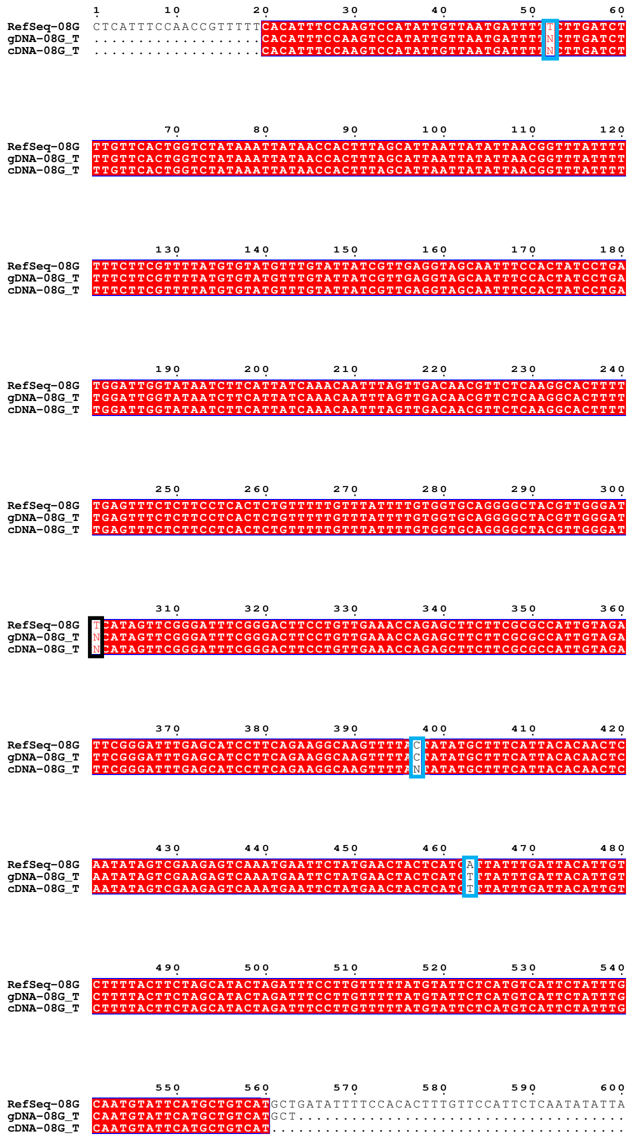

**Locus H08**

Targeted SNP is indicated by black box. Other SNPs are indicated by blue boxes.

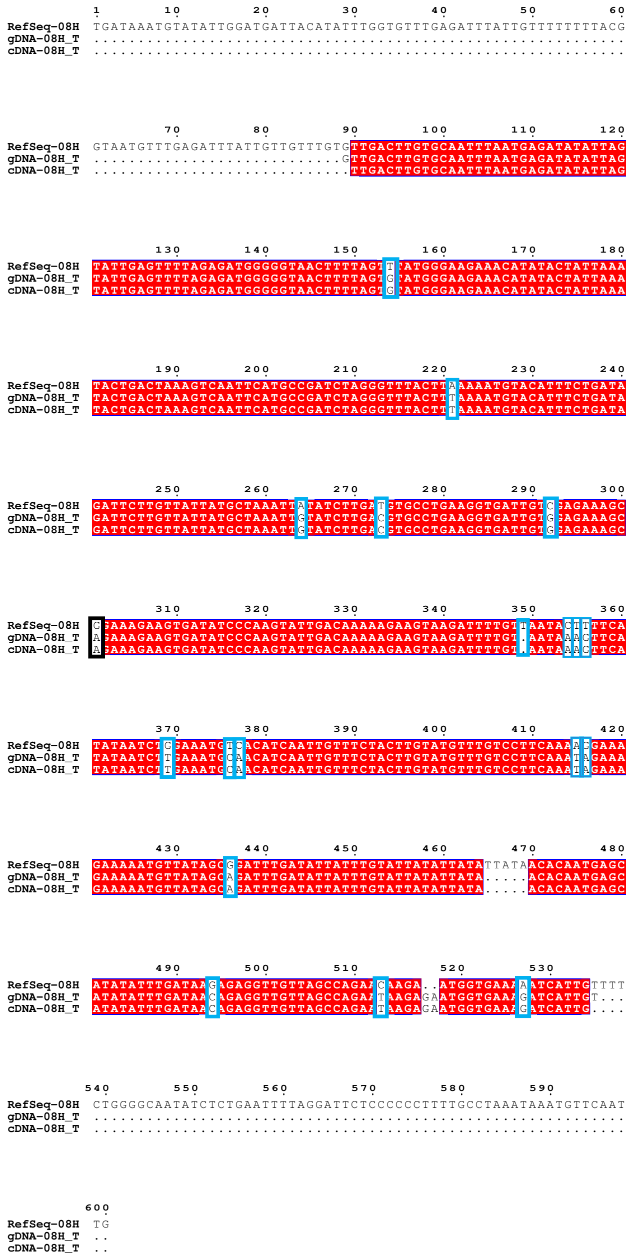

**Locus B09**

Targeted SNP is indicated by black box. Other SNPs are indicated by blue boxes.

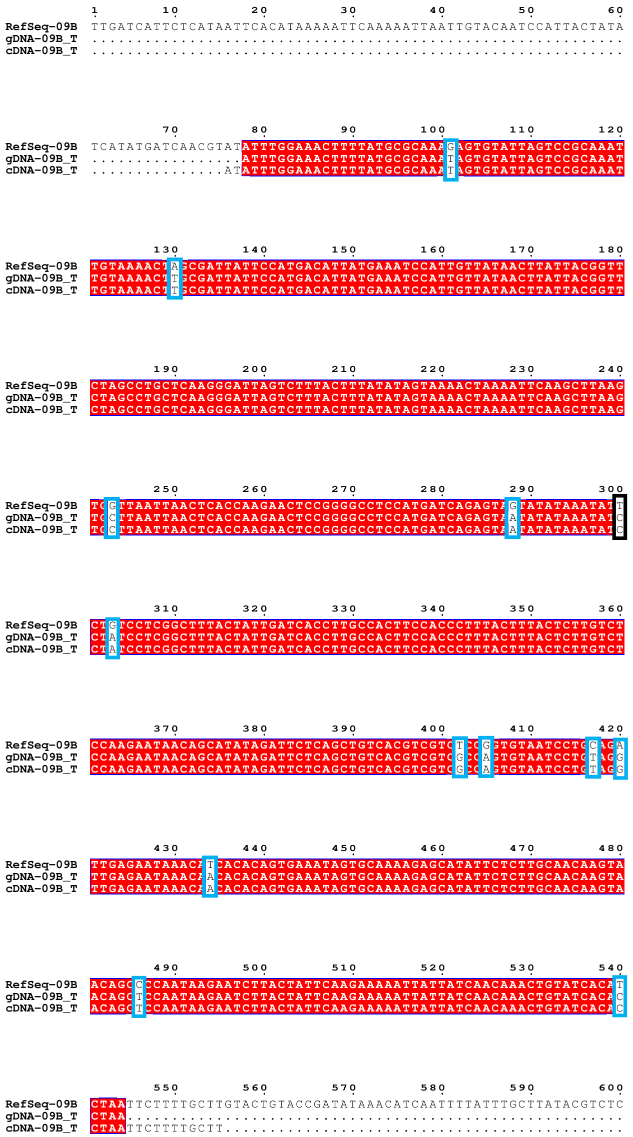

**Locus D09**

Targeted SNP is indicated by black box. Other SNPs are indicated by blue boxes.

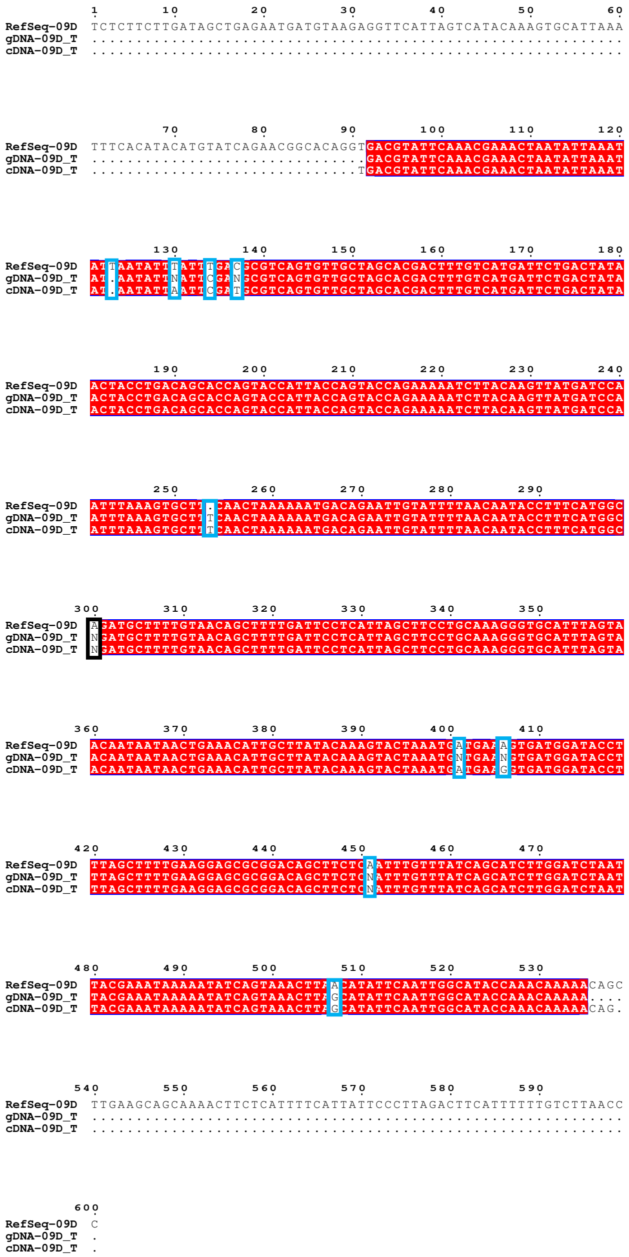

## Locus E09

Targeted SNP is indicated by black box. Other SNPs are indicated by blue boxes.

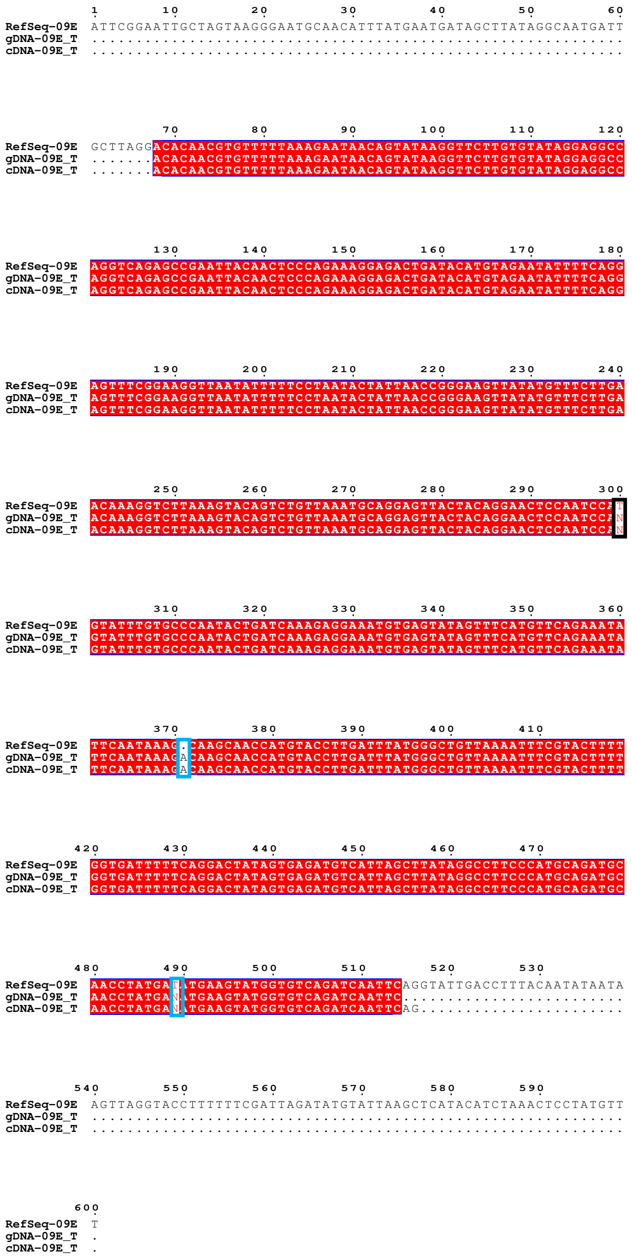

**Locus F09**

Targeted SNP is indicated by black box. Other SNPs are indicated by blue boxes.

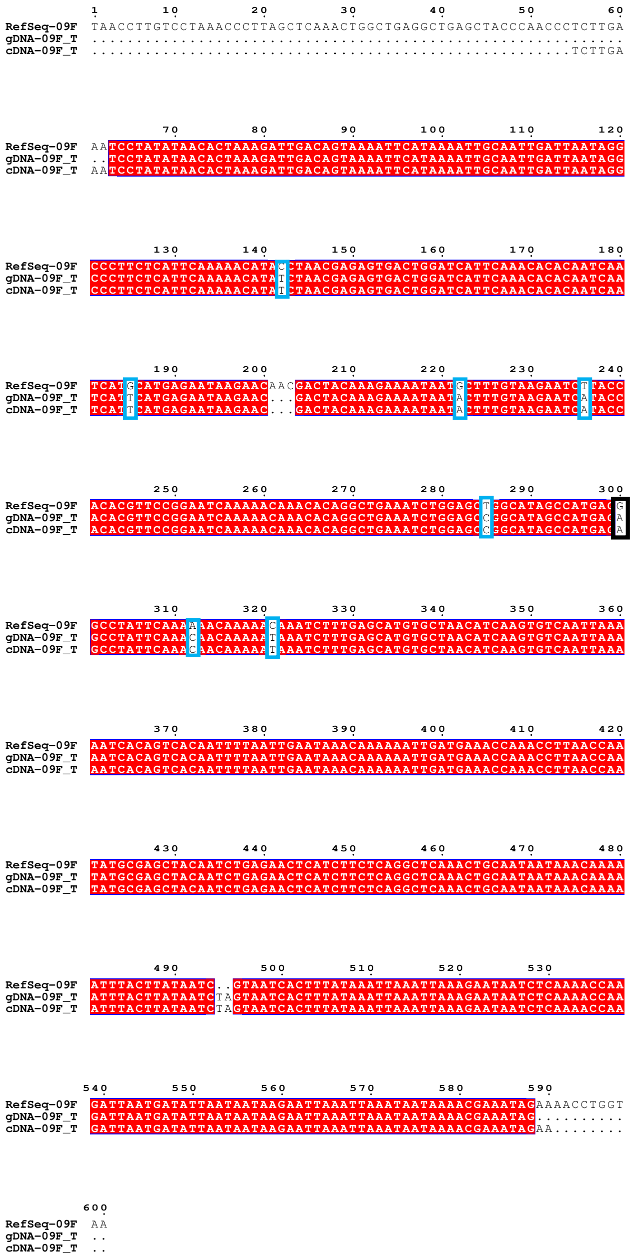

**Locus H09**

Targeted SNP is indicated by black box. Other SNPs are indicated by blue boxes.

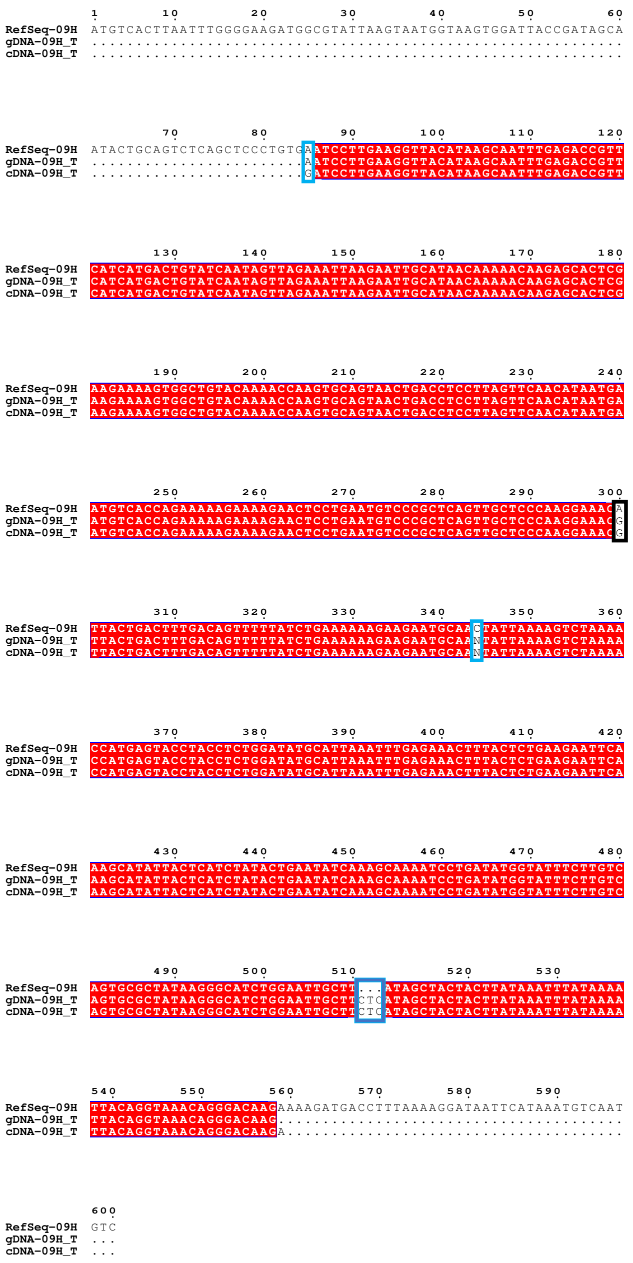

**Locus B10**

Targeted SNP is indicated by black box. Other SNPs are indicated by blue boxes.

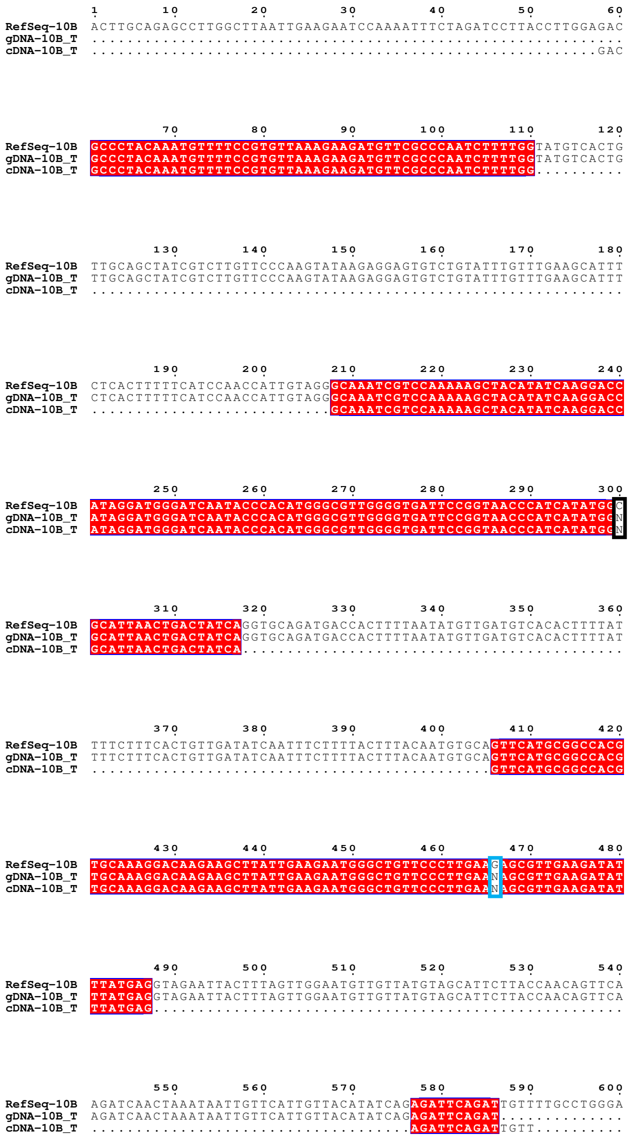

## Locus E10

Targeted SNP is indicated by black box. Other SNPs are indicated by blue boxes.

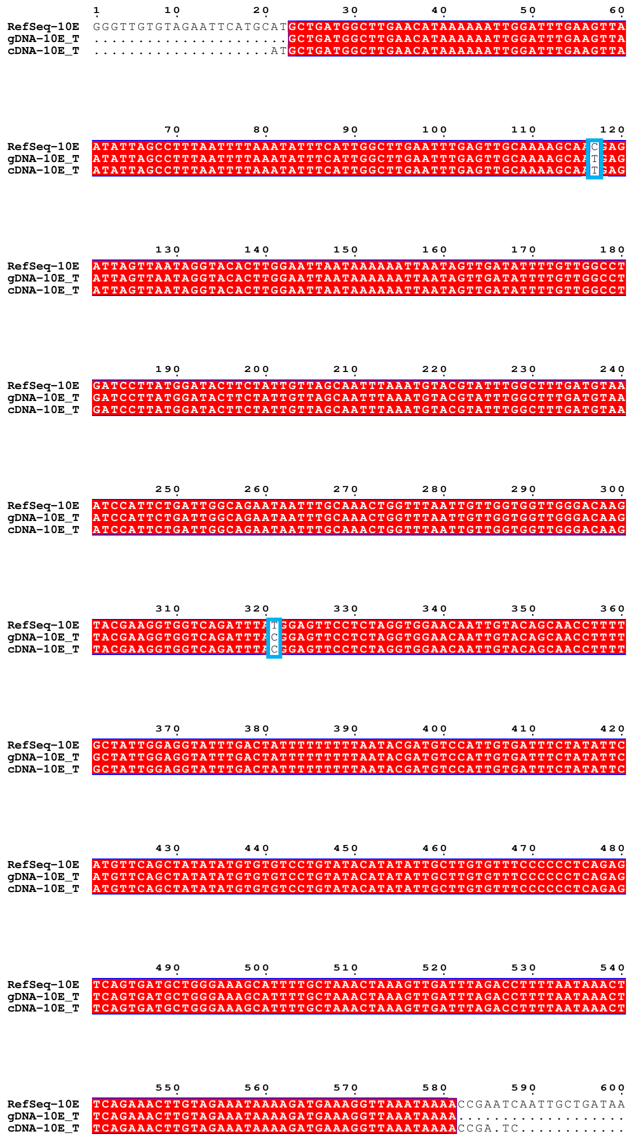

**Locus F10**

Targeted SNP is indicated by black box. Other SNPs are indicated by blue boxes.

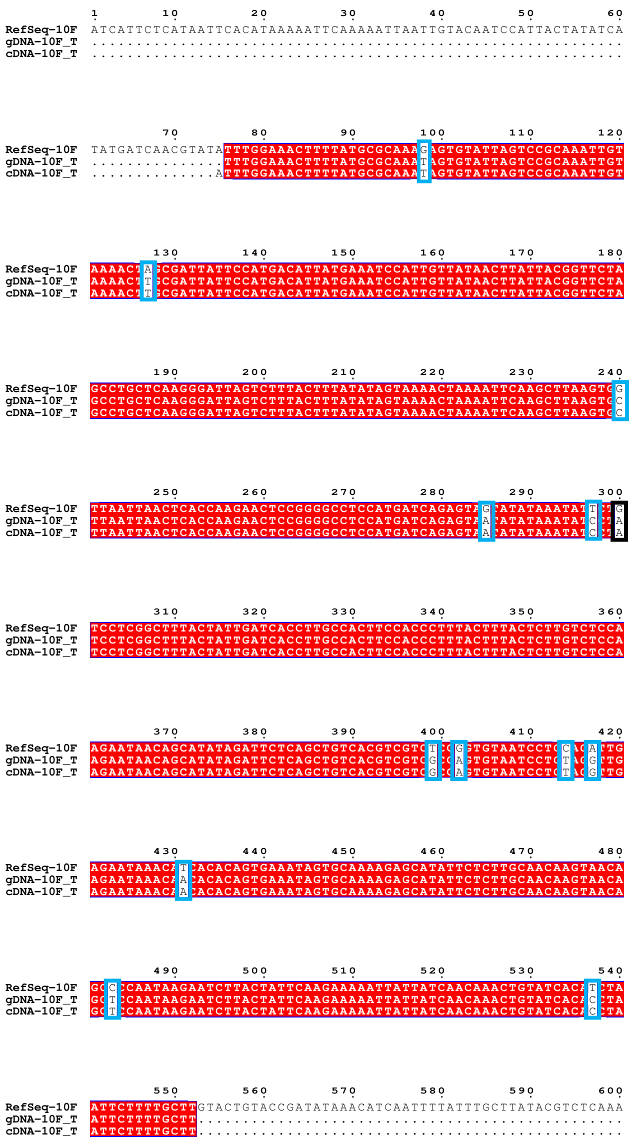

**Locus G10**

Targeted SNP is indicated by black box. Other SNPs are indicated by blue boxes.

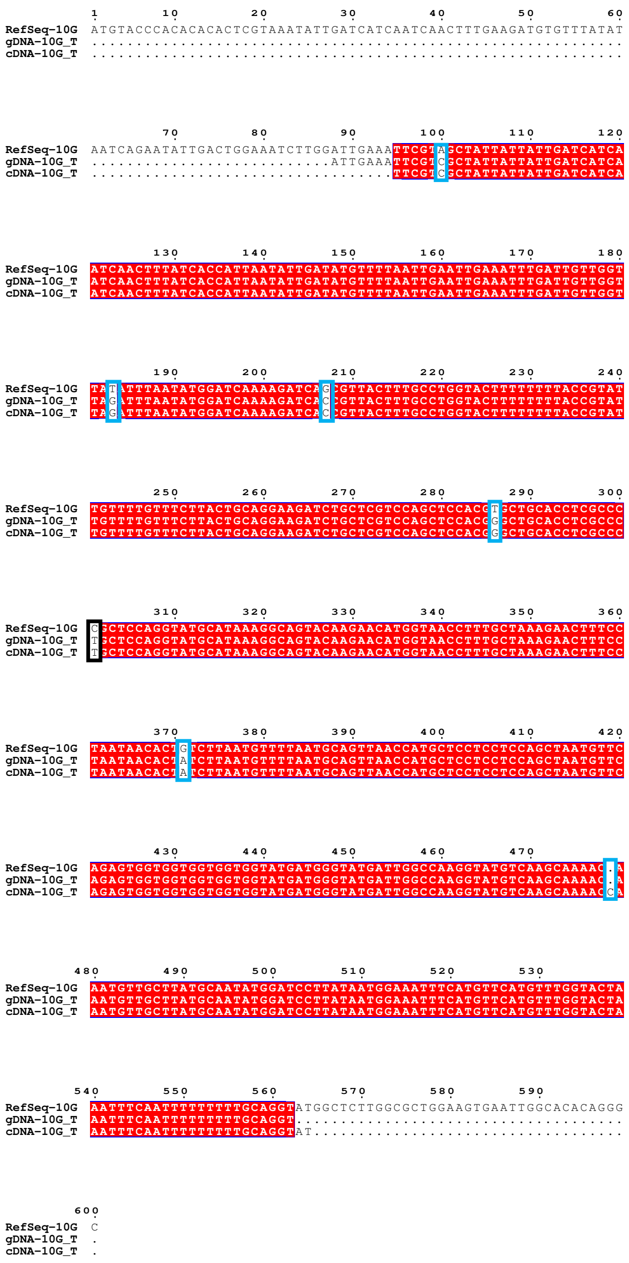

**Locus H10**

Targeted SNP is indicated by black box. Other SNPs are indicated by blue boxes.

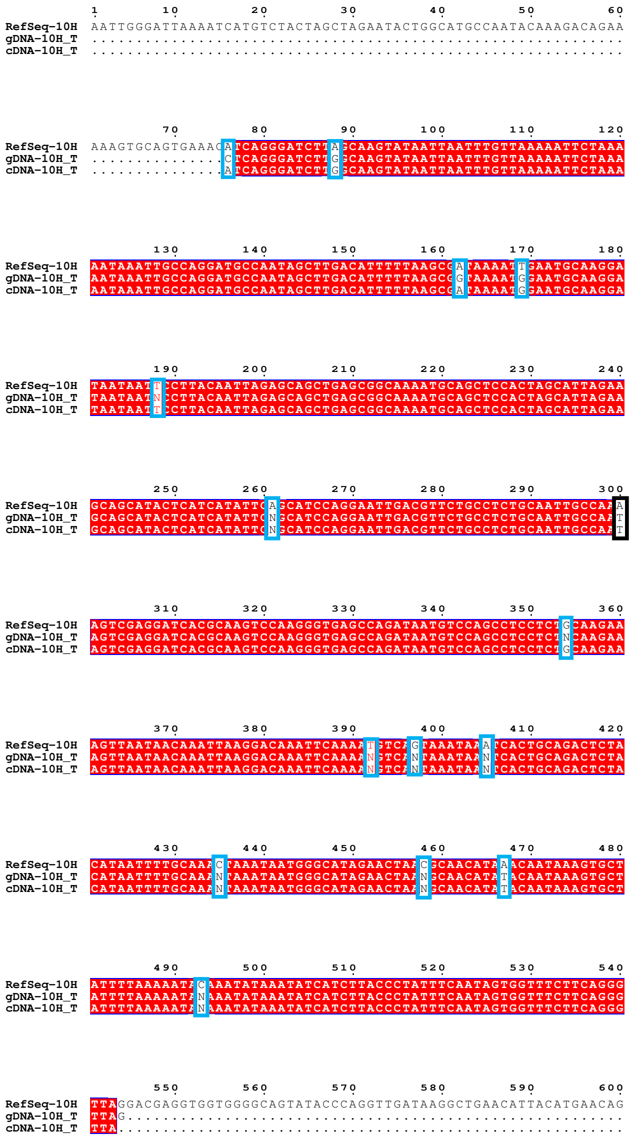

## Locus A11

Targeted SNP is indicated by black box. Other SNPs are indicated by blue boxes.

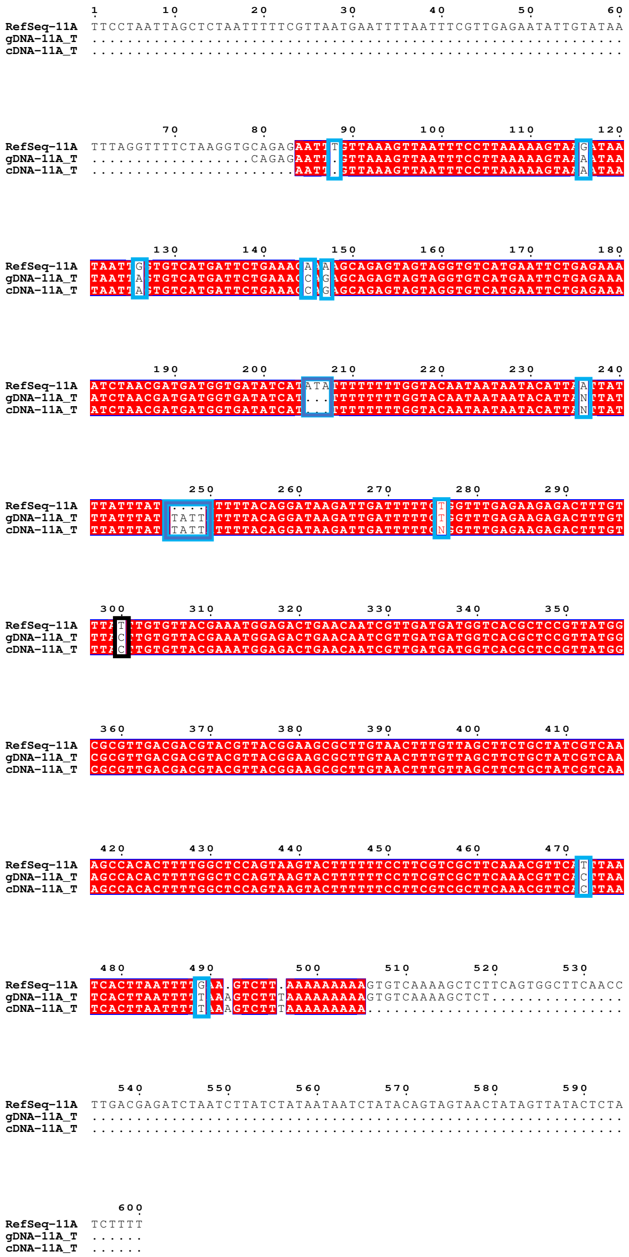

**Locus F11**

Targeted SNP is indicated by black box. Other SNPs are indicated by blue boxes.

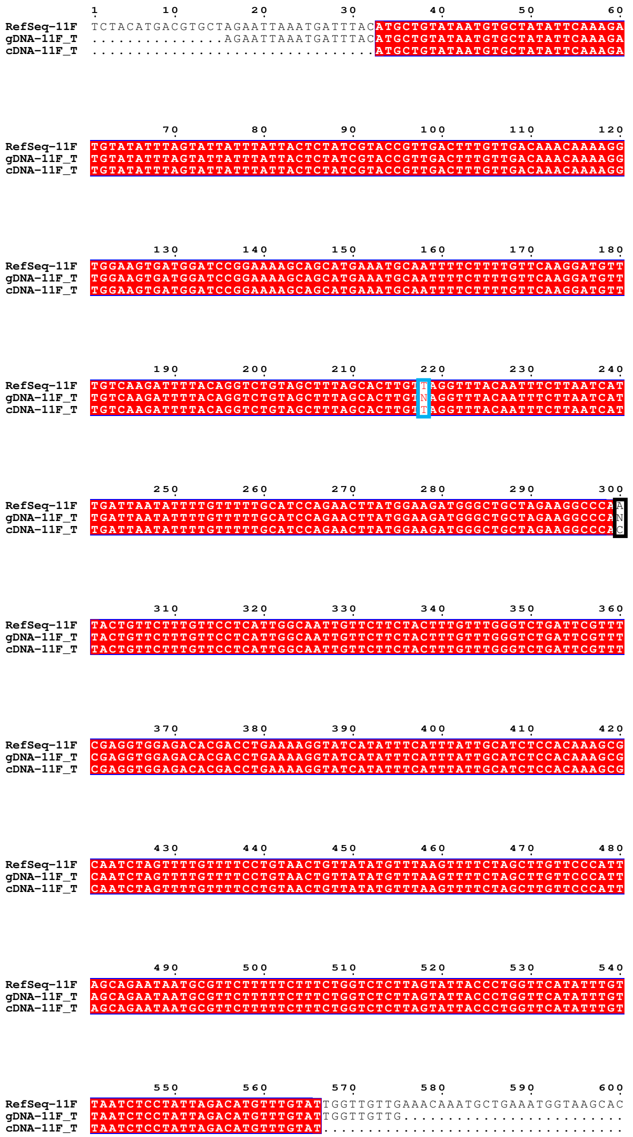

**Locus A12**

Targeted SNP is indicated by black box. Other SNPs are indicated by blue boxes.

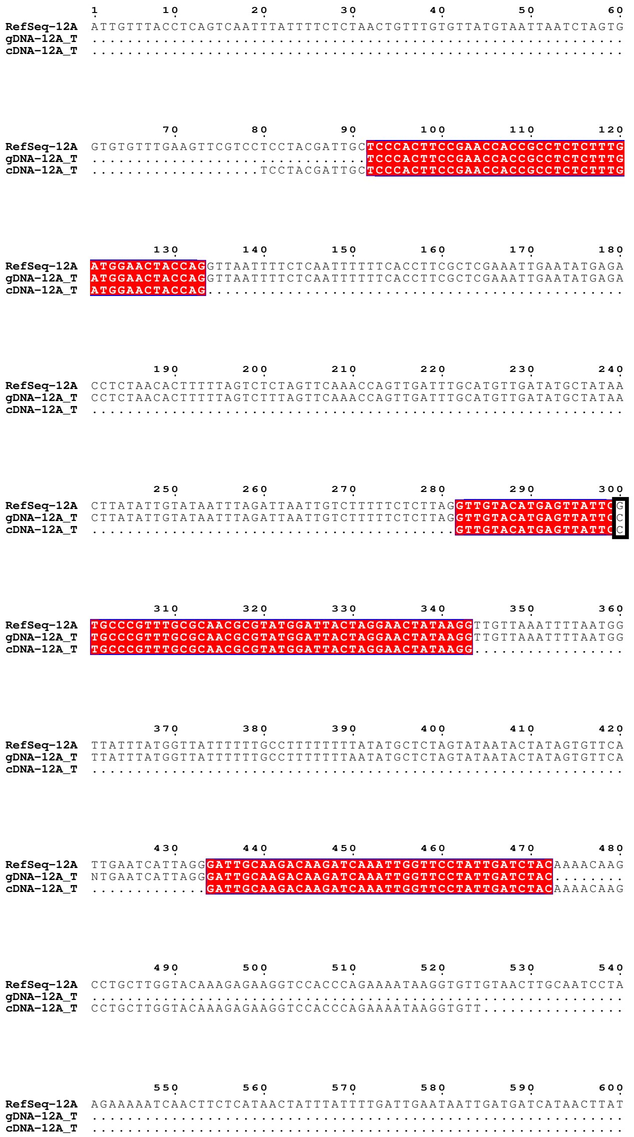

## Locus B12

Targeted SNP is indicated by black box. Other SNPs are indicated by blue boxes.

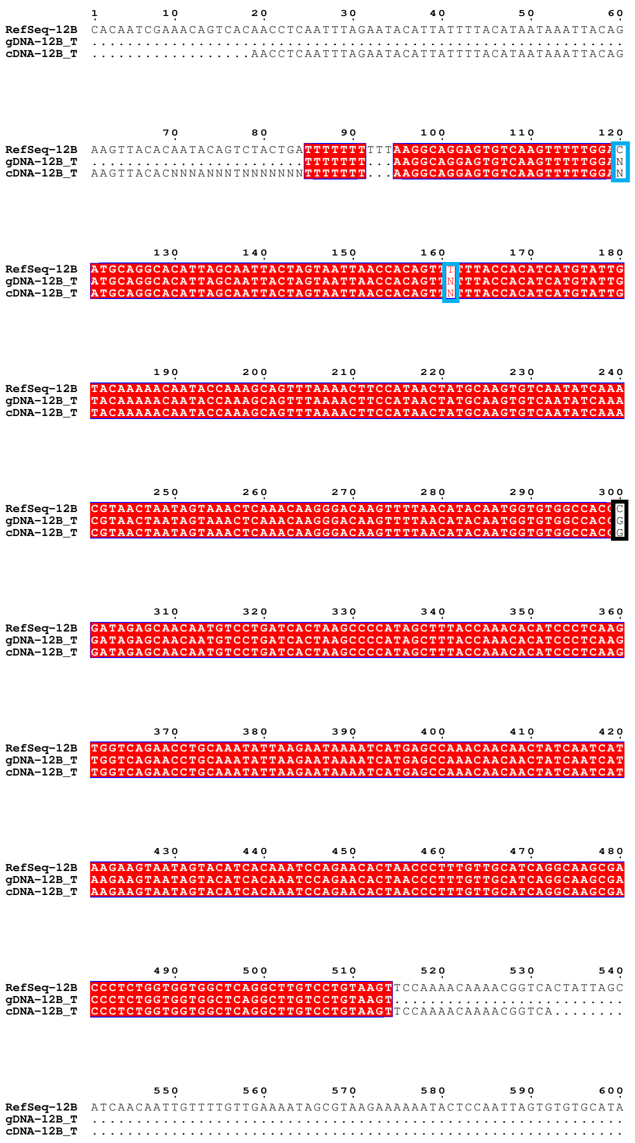

Supplement: Supplemental File 3 — Sequence alignment of RNA-seq identified SNPs. The alignment for each locus included sequences from the following three sources: reference sequence (with RefSeq in the label), sequence generated using genomic DNA (with gDNA in the label), sequence generated using cDNA (with cDNA in the label). doi: 10.6084/m9.figshare.9971879 [file DataSheet_3.pdf]
